# Perceived experts are prevalent and influential within an antivaccine community on Twitter

**DOI:** 10.1093/pnasnexus/pgae007

**Published:** 2024-02-07

**Authors:** Mallory J Harris, Ryan Murtfeldt, Shufan Wang, Erin A Mordecai, Jevin D West

**Affiliations:** Department of Biology, Stanford University, Stanford, CA 94305, USA; Center for an Informed Public, University of Washington, Seattle, WA 98195, USA; Information School, University of Washington, Seattle, WA 98105, USA; Information School, University of Washington, Seattle, WA 98105, USA; Department of Biology, Stanford University, Stanford, CA 94305, USA; Department of Biology, Stanford University, Stanford, CA 94305, USA; Center for an Informed Public, University of Washington, Seattle, WA 98195, USA

**Keywords:** antivaccine, public health, social media, misinformation

## Abstract

Perceived experts (i.e. medical professionals and biomedical scientists) are trusted sources of medical information who are especially effective at encouraging vaccine uptake. The role of perceived experts acting as potential antivaccine influencers has not been characterized systematically. We describe the prevalence and importance of antivaccine perceived experts by constructing a coengagement network of 7,720 accounts based on a Twitter data set containing over 4.2 million posts from April 2021. The coengagement network primarily broke into two large communities that differed in their stance toward COVID-19 vaccines, and misinformation was predominantly shared by the antivaccine community. Perceived experts had a sizable presence across the coengagement network, including within the antivaccine community where they were 9.8% of individual, English-language users. Perceived experts within the antivaccine community shared low-quality (misinformation) sources at similar rates and academic sources at higher rates compared to perceived nonexperts in that community. Perceived experts occupied important network positions as central antivaccine users and bridges between the antivaccine and provaccine communities. Using propensity score matching, we found that perceived expertise brought an influence boost, as perceived experts were significantly more likely to receive likes and retweets in both the antivaccine and provaccine communities. There was no significant difference in the magnitude of the influence boost for perceived experts between the two communities. Social media platforms, scientific communications, and biomedical organizations may focus on more systemic interventions to reduce the impact of perceived experts in spreading antivaccine misinformation.

**Significance Statement**

Despite several high-profile examples of perceived experts spreading vaccine misinformation, no study has systematically surveyed the size and influence of the group of antivaccine perceived experts. On Twitter, perceived experts had a sizable presence in the set of users arguing COVID-19 vaccines are unsafe and ineffective, where they shared misinformation and academic sources. Perceived experts may be important antivaccine influencers, as they were overrepresented in central network positions and were significantly more likely to receive engagements compared to perceived nonexperts. The influence boost for perceived experts in the antivaccine and provaccine communities were not significantly different.

## Introduction

Vaccine refusal poses a major threat to public health and has been a particular concern during the COVID-19 pandemic (1–4). An estimated 232,000 vaccine-preventable COVID-19 deaths occurred in unvaccinated adults in the United States across a 15-month period (May 2021–September 2022) (5). Exposure to misinformation (i.e. false or misleading claims) may reduce vaccine uptake, increase individual risk of morbidity and mortality, and potentially lead to disease outbreaks (6–8). The internet, particularly social media, is an important source of both vaccine information and misinformation (3, 7, 9–11). Social media surveillance has been proposed as a strategy to assess public opinion about vaccination and to study patterns in vaccine decision-making that may inform interventions (4, 12–15). For example, online social networks often contain *influencers*, users who play outsized roles in information propagation and receive significantly more engagement with their content than other users (16–20). Once identified, influencers may be targeted to optimize rapid dissemination of information (e.g. public service announcements or fact checks) (16, 20–22) or to reduce the propagation of harmful content with minimal intervention (18, 23, 24).

Information consumers often use markers of credibility to assess different sources (25, 26). Specifically, *prestige bias* describes a heuristic where one preferentially learns from individuals who present signals associated with higher status (e.g. educational and professional credentials) (27–29). Importantly, prestige-biased learning relies on signifiers of expertise that may or may not be accurate or correspond with actual competence in a given domain (25, 30). Therefore, we will refer to *perceived experts* to denote individuals whose profiles contain signals that have been shown experimentally to increase the likelihood that an individual is viewed as an expert on COVID-19 vaccines (31), although credentials may be misrepresented or misunderstood (user profiles may be deceptive or ambiguous, audiences may not understand the domain specificity of expertise, and platform design may impair assessments of expertise if partial profile information is displayed alongside posts). We focus on the understudied role of perceived experts as potential antivaccine influencers who accrue influence through prestige bias (4, 13). Medical professionals, biomedical scientists, and organizations are trusted sources of medical information who may be especially effective at persuading people to get vaccinated and correcting misconceptions about disease and vaccines (29, 32–35), suggesting that prestige bias may apply to vaccination decisions, including for COVID-19 vaccines (36, 37).

Despite the large body of research on perceived experts who recommend vaccination, the prevalence and influence of perceived experts acting in the opposite role, as disseminators of false and misleading claims about health has not been studied directly. Prior work on antivaccine influencers suggested a category analogous to our definition of perceived experts and provided notable examples (2, 38–40). For example, former physician Andrew Wakefield and other perceived experts promulgated the myth that the measles, mumps, and rubella (MMR) vaccine causes autism (10), perceived experts appeared in the viral Plandemic conspiracy documentary and other antivaccine films (41–43), and 6 of the 12 antivaccine influencers identified as part of the “Disinformation Dozen” responsible for a majority of antivaccine content on Facebook and Twitter included medical credentials in their social media profiles (44). Antivaccine users comprised a considerable proportion of apparent medical professionals on Twitter (a subset of perceived experts excluding scientific researchers) sampled based on use of a particular hashtag (45) or inclusion of certain keywords in their profiles (46, 47). The number and population share of perceived experts in groups opposing COVID-19 vaccines on the microblogging website Twitter has not been assessed systematically, an important step toward understanding the scale of this set of potential antivaccine influencers and one of the goals of this article. We ask (RQ1): How many perceived experts are there in the antivaccine and provaccine communities?

In addition to signaling expertise in their profiles, perceived experts may behave like biomedical experts by making scientific arguments and sharing scientific links but also propagate misinformation by sharing unreliable sources. Antivaccine films frequently utilize medical imagery and emphasize the scientific authority of perceived experts who appear in the films (42, 43, 48). Although vaccine opponents reject scientific consensus, many still value the brand of science and engage with peer reviewed literature (49). Scientific articles are routinely shared by Twitter users who oppose vaccines and other public health measures (e.g. masks), but sources may be presented in a selective or misleading manner (40, 49–53). At the same time, misinformation claims from sources that often fail fact checks (i.e. low-quality sources) are pervasive within antivaccine communities, where they may exacerbate vaccine hesitancy (6, 18, 54, 55). To understand the types of evidence perceived experts use to support their arguments, we ask (RQ2): How often do perceived experts in the antivaccine community share misinformation and academic sources relative to other users?

After describing the types of information that perceived experts share, we evaluate their ability to reach large audiences who help spread their messages. Various network centrality metrics describe the importance of a given user (node) to information flow based on connections to other users (Table S1). Centrality metrics are commonly used to rank the importance of different users within a social media network and help identify influential users (17, 56–59). Provaccine perceived experts were highly central in other Twitter networks discussing vaccines, but no prior analysis has focused on the the centrality of perceived experts in the antivaccine community (60, 61). In addition to a user’s centrality to the whole network, its ability to span opposing communities may be particularly significant (62). “Cognitive bridges,” or users who share content of interest to both antivaccine and provaccine communities may be particularly important due to their potential to connect vaccine skeptics with accurate information or to reduce vaccine confidence in provaccine audiences (53). If perceived experts occupy central and bridging network positions, they may be well-positioned to share their opinions with other users and share (mis)information about vaccines. We therefore ask (RQ3): Do perceived experts occupy key network positions (i.e. as central and bridging users)?

In general, perceived expertise may increase user influence within the provaccine and antivaccine communities. Although vaccine opponents express distrust in scientific institutions and the medical community writ large, they simultaneously embrace perceived experts who oppose the scientific consensus as heroes and trusted sources (10, 41, 43, 48). Medical misinformation claims attributed to perceived experts were some of the most popular and durable topics within misinformation communities on Twitter during the COVID-19 pandemic (12, 63). In fact, compared to individuals who agree with the scientific consensus, individuals who hold counter-consensus positions may actually be more likely to engage with perceived experts that align with their stances (51, 64, 65). This expectation is based on experimental work on *source-message incongruence*, which suggests that messages are more persuasive when they come from a surprising source (66–68). This phenomenon may extend to the case where perceived experts depart from the expected position of supporting vaccination. In an experiment where participants were presented with claims from different sources about a fictional vaccine, messages from doctors opposing vaccination were especially influential and were transmitted more effectively than provaccine messages from doctors (69). However, this effect has not been tested for actual perceived experts commenting on real vaccines. By quantifying the relative impact of perceived experts within the antivaccine community compared to other individuals, we will establish whether they represent a particularly influential group that should be specifically considered in interventions to encourage vaccine uptake. We ask (RQ4): Are perceived experts more influential than other individual users? We test whether perceived experts experience an influence boost in both the antivaccine community (H1) and the provaccine community (H2). We additionally hypothesize that perceived experts experience a larger influence boost within the antivaccine community compared to the provaccine community due to source-message incongruence (H3).

## Results

### Perceived experts are present throughout the coengagement network, including in the antivaccine community

For this study, we collected over 4.2 million unique posts to Twitter containing keywords about COVID-19 vaccines during April 2021. We constructed a coengagement network where users were linked if they their posts were retweeted (shared) at least 10 times by at least two of the same users, meaning that they shared an audience (Fig. 1) (62). In Section S10 (Figs. S23–S39), we show that findings are robust to parameters used to define edges in the coengagement network.

**Fig. 1.**
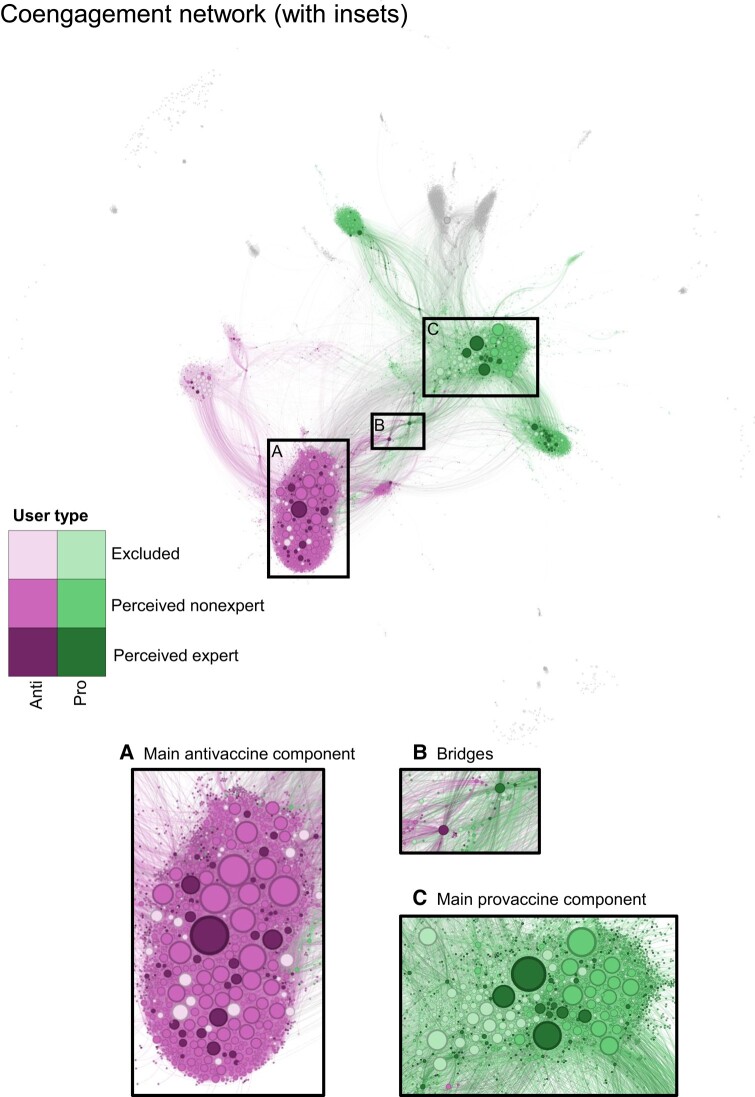
The coengagement network of users tweeting about COVID-19 vaccines is divided into two large communities. Users are represented as circles and scaled by degree centrality. Edges connect users that were retweeted at least 10 times by at least two of the same users. We highlight the two largest communities detected using the Infomap algorithm: antivaccine (pink, lower left) and provaccine (green, top right). Shades indicate account type: excluded from analyses (light); individual perceived nonexpert (medium); and perceived expert (dark). Users outside of the two largest communities are gray. Insets provide more detailed views of: A) the main component of the antivaccine community, B) bridges between the provaccine and antivaccine communities, and C) the main component of the provaccine community. Each edge is colored based on the color of one of the two users it connects, randomly selected. A higher-resolution image without annotations is available as Fig. S1.

The coengagement network consisted of 7,720 accounts linked by 72,034 edges (Fig. 1). Five thousand one-hundred and seventy-one of those accounts had English language profiles and corresponded to individuals. Twenty-four of the individual users with English profiles added or removed expertise cues in their profiles over the course of the study and were thus excluded from the analysis. Of the remaining 5,147 accounts, 13.1% (678 users) were perceived experts. Perceived experts rarely provided cues of expertise in their name alone. Instead, almost all users indicated expertise in their description, with approximately half of users including expertise cues in both their name and description (Fig. S2). There was substantial agreement between coders on whether a user was in an excluded category, a perceived nonexpert, or a perceived expert (κ=0.687, measured on a sample of 500 accounts).

The coengagement network was separated into two main communities (i.e. densely connected groups of users) using the Infomap community detection algorithm. One generally expressed a negative stance toward COVID-19 vaccines while users in the other were primarily positive, leading us to label the communities as antivaccine and provaccine, respectively. The two largest communities contained 79.6% of total accounts and 66.1% of English-language individual accounts in the network. Stance toward COVID-19 vaccines in popular tweets by users with the greatest degree centrality was relatively consistent, meaning that very few users posted a combination of tweets that were positive and negative in stance, although most users posted some neutral tweets as well (Fig. S3). Further, stance was generally shared within communities; popular nonneutral tweets by central users in the antivaccine and provaccine communities almost exclusively expressed negative and positive stances, respectively, with a few exceptions. Although there was moderate inter-rater reliability between coders on whether individual tweets were negative, positive, or neutral about vaccines (κ=0.567), there was high agreement on the overall stance of each user (κ=0.805 for whether a given user posted more antivaccine tweets, provaccine tweets, or equal numbers of both).

The provaccine community was larger than the antivaccine community (3,443 and 2,704 users, respectively). Perceived experts were present in both communities, but constituted a larger share of individual users in the provaccine community (17.2% or 386 accounts) compared to the antivaccine community (9.8% or 185 accounts) (Fig. 3). Both communities were further subdivided into subcommunities corresponding to language and geographic focus (Fig. S7 and Table S4).

### Perceived experts in the antivaccine community share both low-quality and academic sources

**Fig. 2.**
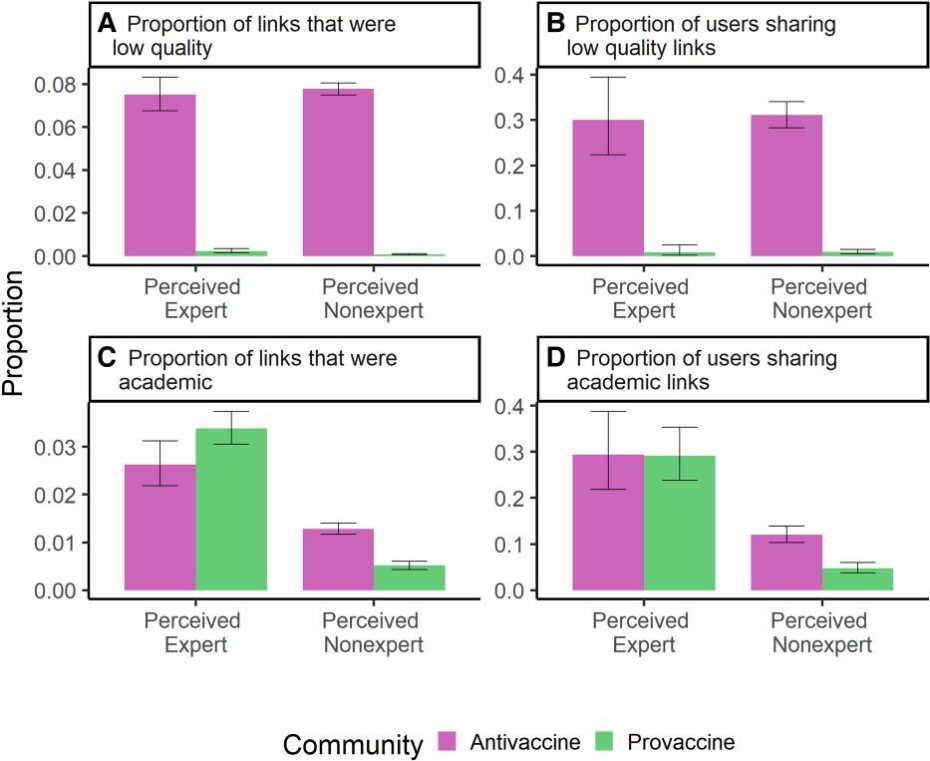
Users in the antivaccine community share low-quality sources and perceived experts share academic research. Each panel compares a different metric of link-sharing by perceived experts and perceived nonexperts in the antivaccine (left bar in each pair, pink) and provaccine (right bar in each pair, green). The metrics are: A) proportion of checked links that were from low-quality sources, B) proportion of users that shared at least one low-quality source, C) proportion of checked links that were from academic research sources, and D) proportion of users that shared at least one academic research source. 95% binomial proportion CIs are indicated by black error bars. Differences between users in the antivaccine vs. provaccine community sharing low-quality sources were significant (P<0.001 at both the link and user levels), and differences between perceived experts and perceived nonexperts sharing academic sources were significant (P<0.001 at both the link and user levels).

We found marked differences in sharing of low-quality and academic sources depending on community and perceived expertise (Fig. 2). For both types of sources, we calculated a user-level metric (the proportion of users who shared at least one link of a given type; Fig. 2B and D) and a link-level metric (the proportion of all links that were of a given type; Fig. 2A and C). Perceived experts posted more frequently and included links in a greater proportion of their posts (Fig. S4).

Compared to the provaccine community, perceived experts and perceived nonexperts in the antivaccine community shared low-quality sources at significantly greater rates (P<0.001 for proportion of links and users). Low-quality sources were almost exclusively shared in the antivaccine community, although low-quality sources generally comprised a relatively small proportion of assessed links (Fig. 2A and B). Many users in the antivaccine community shared at least one low-quality link (30% of perceived experts and 31% of perceived nonexperts) compared to about 0.8% of users in the provaccine community (3 perceived experts and 14 perceived nonexperts) (Fig. 2B).

In both communities, perceived experts shared academic research links at a significantly greater rate compared to perceived nonexperts (Fig. 2C and D) (P<0.001 for proportion of links and users). Among perceived nonexperts, there was significantly greater academic link-sharing in the antivaccine community (P<0.001 for proportion of links and users). About 10% of perceived experts in the antivaccine community shared both academic and low-quality sources, while approximately 20% shared only academic or only low-quality sources (Fig. S5). Right-biased partisan sources were significantly more prevalent in the antivaccine community, but there was no significant difference between communities in propensity to share left-biased partisan sources (Fig. S6).

### Perceived experts are overrepresented as central and bridging users

**Fig. 3.**
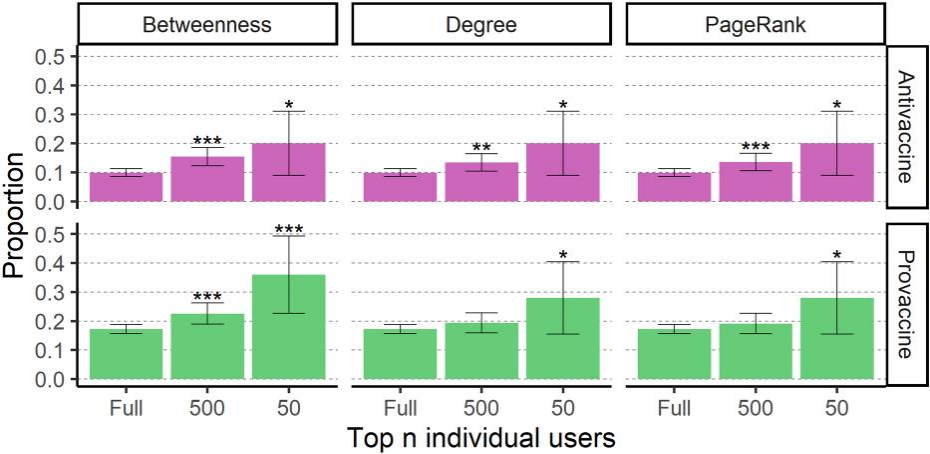
Perceived experts are overrepresented as the most central users in the provaccine and antivaccine communities. Plots are arranged in a grid where each row corresponds to users in one of the two largest communities: the antivaccine and provaccine communities (top in pink and bottom in green, respectively). Each column corresponds to a different centrality metric: betweenness (left), degree (middle), and PageRank (right). The *x*-axis indicates the size of each subset (*n*), corresponding to the full community, and the 500 or 50 users with the greatest centrality for each metric. Bar height indicates the proportion of users in each subset that are perceived experts and error bars give 95% binomial proportion CI. Stars above the error bars indicate whether perceived experts are significantly overrepresented within a given group of central users (*P<0.05, **P<0.01, ***P<0.001).

**Fig. 4.**
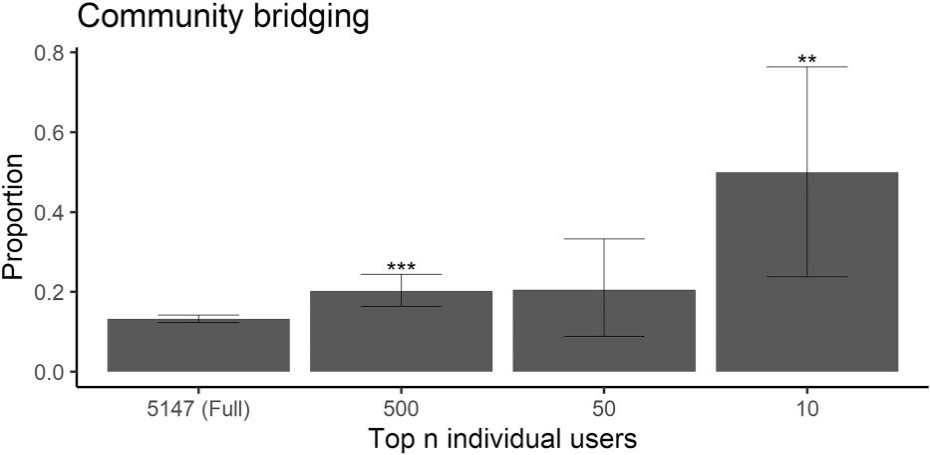
Perceived experts disproportionately act as key bridges between the provaccine and antivaccine communities. The *x*-axis indicates the size of each subset (*n*), corresponding to the full population of 5,147 (all individual users as a basis of comparison), and the 500, 50, or 10 users with the greatest community bridging score. Bar height indicates the proportion of users in each population sample that are perceived experts and error bars give 95% CIs for the proportions. Stars above the error bars indicate whether perceived experts are significantly overrepresented within a given group of bridging users (**P<0.05, **P<0.01, ***P<0.001).

Although perceived experts represented a relatively small share of the individual users in the coengagement network, they disproportionately occupied important positions in the network as central and bridging users (Figs. 3 and 4). Perceived experts were overrepresented among users with the greatest betweenness, degree, and PageRank centrality in both communities (Fig. 3; see Table S1 for an explanation of centrality metrics in the coengagement context).

Perceived experts in both communities were highly overrepresented among the 500 users with the greatest betweenness centrality (P<0.001 for both communities) and overrepresented among the 50 users with the greatest betweenness centrality by a factor of about two (P=0.014 and P<0.001 for the antivaccine and provaccine communities, respectively) (Fig. 3).

Ranking on degree and PageRank centrality, perceived experts were more strongly overrepresented as central users in the antivaccine community compared to the provaccine community (Fig. 3). Perceived experts in the antivaccine community were about two times more prevalent in the group of central users (20% of the 50 top users ranked on both metrics) compared to their share of the population, while perceived experts in the provaccine community were overrepresented by a factor of 1.6. By both metrics, perceived experts in the antivaccine community were significantly overrepresented in the 500 most central users (P=0.001 and P<0.001 for degree and PageRank centrality, respectively) and the 50 most central users (P=0.014 for both degree and PageRank centrality). In the provaccine community, perceived experts were significantly overrepresented in the 50 most central users (P=0.032 for both degree and PageRank centrality) but not in the 500 most central users (P=0.1038 and P=0.082).

Perceived experts were significantly overrepresented in the groups of the 500 and 10 top bridges between the provaccine and antivaccine communities (P<0.001 and P=0.001, respectively), but not significantly overrepresented in the 50 top bridges (P=0.09) (Fig. 4). About 20% of the top 500 and 50 users ranked by bridging scores were perceived experts, and five of the 10 users with the greatest bridging scores were perceived experts (Figs. 1B and 4). There was little variation in which users were highly ranked based on different network metrics (Figs. S9–S12).

### Perceived experts are more influential than other individuals in the antivaccine and provaccine communities

**Fig. 5.**
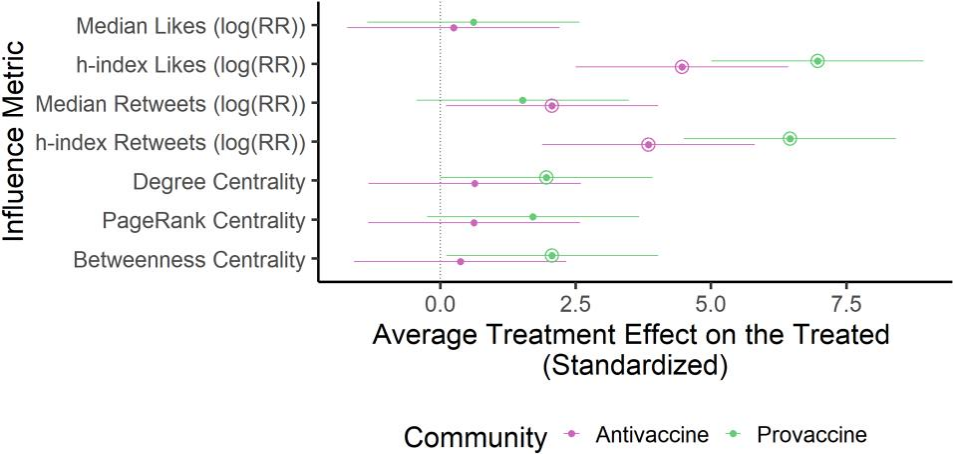
In the antivaccine and provaccine communities, perceived experts receive more engagements compared to other users. For each influence metric (*y*-axis, Table S3), we plot the standardized ATT as a point and corresponding 95% CI. For each metric, the value for the provaccine community is displayed on top (green) and the value for the antivaccine community is displayed on the bottom (pink). Positive values (to the right of the vertical line) indicate an influence boost for perceived experts. Instances where the effects were significantly greater than zero (P<0.05) are indicated with an additional circle around the point estimate. For engagement metrics, we report the natural log of the risk ratio of a given engagement for perceived experts compared to perceived nonexperts.

Using propensity score matching, we achieved excellent balance across matching covariates (Figs. S13–S15). Using these propensity-matched pairs for comparison, we calculated the average treatment effect on the treated (ATT) (i.e. average difference in influence between perceived experts and perceived nonexperts) across influence metrics based on engagements and centrality (Table S3). Perceived experts received more engagements (likes and retweets) on their posts in both communities (based on h-index metrics, which also account for the number of posts by a user that received many engagements) and had greater betweenness and degree centrality than other individual users in the provaccine community only (Fig. 5 and Tables S6–S8).

Perceived experts in the antivaccine community were 1.43 (95% CI 1.02–1.99) times more likely to receive retweets on their median post than would be expected if they were not perceived experts (Fig. 5 and Table S6). There was no significant effect of perceived expertise on median likes, but there was a significant effect of perceived expertise on h-index for likes and retweets (Fig. 5 and Table S6). Perceived expertise did not significantly affect centrality (betweenness, degree, and eigenvector) within the antivaccine community *on average* (Fig. 5 and Table S6), although we found in the previous section that perceived experts were overrepresented in the *tail* of the distribution as highly central users compared to the full (unmatched) set of perceived nonexperts (see Section S9 and Fig. S19).

Perceived experts in the provaccine community had a significant positive on engagement metrics based on the h-index, but not metrics based on median, and additionally had a significant positive ATT for betweenness and degree centrality (Fig. 5 and Table S7). Although perceived experts in the provaccine community tended to have a greater ATT across all influence metrics than those in the antivaccine community, the differences in ATT between groups were not statistically significant for any influence metrics (Figs. S16 and 5 and Table S8). Matching results were generally robust to matching specifications (Figs. S20–S22) and parameters defining edges in the coengagement network (Figs. S36 and S37). However, as explained in Sections 9 and 10, there were some differences in which ATTs were significant depending on matching specifications and coengagement network parameters. There was also a significantly greater boost in median retweets for perceived experts in the antivaccine community compared to the provaccine community in coengagement networks constructed using different parameters (Figs. S38 and S39).

## Discussion

The antivaccine community contains its own set of perceived experts. These perceived experts represent 9.8% of individual users within a large antivaccine community on Twitter, comprising a substantial group that extends beyond the handful of high-profile antivaccine influencers with biomedical credentials who have been noted anecdotally (38, 39). Although surveys have found broad support for COVID-19 vaccination among medical providers (70, 71), 28.9% of perceived experts in the two largest communities of the coengagement network we examined were part of the antivaccine community, a proportion similar to those reported by other studies of COVID-19 vaccine attitudes expressed by medical professionals on Twitter (45–47). Antivaccine perceived experts are therefore overrepresented on Twitter compared to the share of actual biomedical experts who oppose COVID-19 vaccines, which may lead observers to underestimate the scientific consensus in favor of COVID-19 vaccination, in turn reducing vaccine uptake (70, 72, 73).

Within the antivaccine community, perceived experts may combine misinformation with claims that appear scientific. Low-quality sources in the coengagement network were overwhelmingly shared by the antivaccine community, and perceived experts shared low-quality sources at similar rates compared to other individuals (Fig. 2A and B), suggesting that they directly contribute to the widespread misinformation in the antivaccine community noted by other studies (54, 74, 75). Perceived experts, including those in the antivaccine community, performed expertise by sharing and commenting on academic articles at much higher rates than other individual users (Fig. 2C and D). Misinformation claims containing arguments that appear scientific may be particularly effective at reducing vaccine intent (6), suggesting that perceived experts may be responsible for some of the most compelling antivaccine claims.

Perceived experts may also be well-poised to spread their claims online, as they disproportionately occupied key network positions between antivaccine and provaccine communities and within the antivaccine community in the coengagement network (Figs. 3 and 4). Across various centrality metrics, perceived experts were overrepresented in the group of highly central users (Fig. 3), meaning that they reached (and had posts shared by) large and unique audiences. Perceived experts were half of the 10 users with the greatest community bridging scores, meaning that they were shared by audiences for users in both the antivaccine and provaccine communities. Within this set of five perceived experts, four made highly technical arguments in favor of vaccines and corrected potential misunderstandings while the fifth made dramatic claims about vaccine risks. Bridging users could play an important role in changing vaccine stance, although different communities may share distinct subsets of their tweets for substantially different reasons (40, 62). For example, antivaccine audiences may retweet reports of adverse events out of concern that vaccines are unsafe while provaccine audiences may share the same content to emphasize the rarity of such events.

Our hypothesis that perceived experts are, on average, more influential than other users in the antivaccine community was supported by the finding that they received more engagements (i.e. likes and retweets) on their vaccine-related posts than similar users without credentials in their profiles (based on h-index for likes and h-index and median for retweets) (Fig. 5). We also found evidence of this effect in the provaccine community. Perceived experts were significantly more central on average than a matched set of perceived nonexperts in the provaccine community, but this effect was not observed in the antivaccine community (Fig. 5). These findings may be explained by the observations that matching covariates (e.g. follower count and postfrequency) contribute importantly to centrality (Fig. S19) and that overrepresentation of perceived experts in the set of highly central users (i.e. the tail of the distribution) may not be sufficient to significantly increase the mean centrality of perceived experts compared to perceived nonexperts in the antivaccine community (Fig. 3). There was no significant difference in the influence boost for perceived experts between the antivaccine and provaccine communities, contradicting our hypothesis that, due to source-message incongruence, perceived experts hold a greater advantage within the antivaccine community (although we did find weak evidence for this hypothesis based on median retweets in alternative coengagement networks; see Figs. S38 and S39). Overall, these findings suggests that antivaccine audiences do value expert opinion, at least when it confirms their own stances, a result aligned with other studies that have found that scientists and medical professionals are popular sources among vaccine opponents (10, 12, 41, 43, 48, 49, 63).

In sum, this works goes beyond high-profile examples of antivaccine perceived experts to systematically characterize the sizable population of antivaccine perceived experts who had a significant impact on the the Twitter conversation about COVID-19 vaccines in April 2021. While additional work is necessary to determine the robustness of these results across time, location, and setting (including nondigital contexts), our findings have implications for interventions focused on education of medical professionals and the general public. Educational interventions that encourage trust in science could backfire if individuals defer to antivaccine perceived experts who share low-quality sources (76). Instead, education efforts should focus on teaching the public about the scientific process and how to evaluate source credibility to counter the potentially fallacious heuristic of deferring to individual perceived experts (26, 76–78). Although the sample of perceived experts in this study is not representative of the broader community of experts, surveys have found that a nonnegligible minority of medical students and health professionals are vaccine hesitant and believe false claims about vaccine safety (71, 79, 80). Given that perceived experts are particularly influential in vaccine conversations (Figs. 3–5) and that healthcare providers with more knowledge about vaccines are more willing to recommend vaccination, efforts to educate healthcare professionals and bioscientists on vaccination and to overcome misinformation within this community may help to improve vaccine uptake (81).

In addition to helping people evaluate vaccine information, interventions may focus on improving information quality by focusing on communication, social media platform design, and expert community self-governance. Communication efforts by experts recommending COVID-19 vaccines and debunking medical misinformation may help to correct public misunderstandings about expert consensus based on the overrepresentation of antivaccine perceived experts on social media (70). However, perceived experts already constituted one-fifth of individuals in the provaccine community according to our analysis (82–84), and it is not clear whether individual provaccine communicators will be especially persuasive to people who are already engaging with perceived experts who oppose vaccines. Instead, emphasizing the scientific consensus in favor of COVID-19 vaccines and avoiding false balance in communication may help ameliorate misconceptions (70, 85). Further, perceived experts and their professional organizations may build trust and disseminate health information more effectively by developing networked communication strategies to rapidly, openly, and factually address false claims that gain traction while clearly explaining areas of uncertainty and directly addressing legitimate safety concerns (as several of the most central perceived experts in the provaccine community did during the study period, see Fig. S3) (2, 86). User-provided expertise cues were sufficient to garner greater engagement on Twitter in vaccine-related discussions, suggesting that individuals could misrepresent their own credentials to more effectively spread antivaccine misinformation (Fig. 5). Platforms may counter potential deception by establishing mechanisms to verify academic and professional credentials and creating signals within profiles to identify authorities on health-related topics. These measures were implemented on Twitter in the early months of the COVID-19 pandemic but have since been abandoned. Finally, this work illustrates the importance of self-regulation within expert communities, particularly as medical boards clarify that health professionals who spread vaccine misinformation may face disciplinary consequences (87).

### Limitations and extensions

This study relies on proxies for tweet content and user activity that may miss important variation and nuance in stance. Breaking the network into provaccine and antivaccine communities is common across studies of social media networks (54, 55, 60, 74), and stance was generally consistent across popular tweets in either community (Fig. S3), but there were several notable exceptions. Positive tweets from users in the antivaccine community tended to cite vaccine efficacy as an argument against mandating vaccines, while negative tweets from users in the provaccine community (particularly those by perceived experts) praised vaccine regulators for responding to safety signals. Further, sharing a particular misinformation or academic link may not constitute endorsement, particularly in fact-checking contexts. Although academic sources were shared in the antivaccine community, scientific studies may be misrepresented by these users (10, 51, 54) or include articles that have been retracted (40, 50). Future work may examine which academic links were shared within the antivaccine community and how these sources were interpreted. More detailed content analysis may reveal important differences between the communities beyond attitudes toward vaccine (e.g. attitudes toward nonpharmaceutical interventions and perceptions of severity of COVID-19 infection), heterogeneity in vaccine opinion within groups, and specific rhetorical strategies utilized by perceived experts in either group (42, 88, 89). We were unable to assess how many users were exposed to a given tweet, instead relying on engagements as an indicator of tweet popularity, which may lead to us to underestimate the true reach of content. We also could not ascertain how exposure to vaccine-related information in this study influenced health decision-making and behavior, questions that could be directly evaluated in an experimental setting (69). We focused on perceived experts rather than self-proclaimed experts (i.e. experts who intend to be perceived as experts), acknowledging that perceived experts may not realize that they are being viewed as biomedical authorities or wish to be seen as such and that evaluating user intentionality is beyond the scope of this study.

Our analysis is limited to a subset of individuals discussing COVID-19 vaccines. This study is focused on a single month, based on events in the United States and English keywords, constrained to a single coengagement network on Twitter, and limited to conversations that used specific keywords related to COVID-19 vaccination. Initial vaccination capacity, trust in experts, and vaccine uptake vary considerably between countries (8), and our own analysis found considerable geographic clustering across the coengagement network (Fig. S5). Further work may examine how the role of perceived experts in conversations about vaccination differed between regions and across different time periods (including prior to the COVID-19 pandemic and after the emergence of variants of concern with high breakthrough infection rates). Although we excluded the small set of individuals who added or removed signals of expertise from their profile during the study period, examining a longer time period to expand this set of users could enable further analysis of how user behavior and influence changes depending on perceived expertise (90). Twitter users are not representative of the general population (91), and patterns in vaccine conversations on social media do not necessarily reflect actual vaccination trends (13, 15), although vaccine hesitancy may correlate with Twitter activity (7). By filtering the set of accounts considered in this analysis to construct the coengagement network, our findings are further limited to a subset of users who receive repeated engagement from other accounts. Keyword-based methods to collect posts about vaccination may introduce additional biases into the dataset by excluding slang, misspellings, and efforts to evade moderation by using codewords (92). The generalizability of these results should be assessed on different social media platforms (4, 9, 13). The role of experts, particularly those who take counter-consensus positions, is relevant across other scientific topics including climate change, tobacco, and AIDS etiology and treatment (93, 94). These methods could be applied to compare the role of perceived experts in conversations about different scientific and nonscientific topics (e.g. politics and entertainment) to test whether domain specific credentials are necessary to be perceived as an expert and compare the effects of perceived expertise relevant to different conversations (e.g. whether politicians speaking on political matters receive an influence boost comparable to that of medical professionals and scientists discussing COVID-19 vaccines).

## Conclusion

By examining a coengagement network based on Twitter posts in April 2021, we found that the set of antivaccine perceived experts extends far beyond prominent examples noted by others previously (38, 39), suggesting that they should be addressed as a unique and sizable group that blends misinformation with arguments that appear scientific. We also found evidence that perceived experts are more influential than other individuals in the antivaccine community, as they disproportionately occupied central network positions where they could reach large audiences and were significantly more likely to receive engagements on their vaccine-related posts compared to perceived nonexperts (measured by h-index across posts). Perceived experts are not only some of the most effective voices speaking out against vaccine misinformation; they may be some of its most persuasive sources.

## Methods

The Institutional Review Board of Washington University determined that this study (STUDY00017030) was exempt.

### Data collection

Our analysis was conducted on a subset of collections of public tweets related to vaccines and the COVID-19 pandemic, retrieved and stored by the University of Washington’s Center for an Informed Public in real time as they were posted. Tweets that were later deleted, and public tweets from accounts that were suspended or became private are included in the dataset.

We constrained our search period to April 2021 (see Methods S1.1 for further context on the study period). To focus our analysis on content related to COVID-19 vaccines, we selected tweets (i.e. posts) within the collections that mentioned the following keywords related to COVID-19 vaccines: vacc*, vaxx, jab, shot, immuniz*, dose, mrna, pfizer, j&j, jnj, j & j, j n j, johnson and johnson, johnson & johnson, janssen, moderna (where asterisk (*) indicates a wildcard, meaning a set of alphabetical characters of any length). Although this protocol focuses on vaccine administration in the United States and English language content, tweet selection was not constrained to a specific geographic region. In total, we retrieved 4,276,842 unique tweets including quote tweets (when another post is shared with commentary) and replies (a direct response to another user’s tweet) from April. A total of 5,448,314 unique users participated in the Twitter conversation about COVID-19 vaccines during the study period by either posting original content or retweeting (i.e. sharing) another user’s tweet on the topic. We additionally retrieved retweets, quote tweets, and replies linked to tweets in the April collection that were posted within 28 days of the original tweet (extending the dataset to May 28) to compare the number of likes and retweets each tweet in the April study period received across a window of the same length (4 weeks).

We randomly generated a unique numeric identifier for each user to protect user privacy (particularly for users who are not public figures) while allowing our findings to be reproduced (Methods S1.2).

### Coengagement network construction

We examined the activity of influential users with shared audiences by generating a coengagement network. First, we constructed a directed graph where each edge connects a user to another user whom they have retweeted at least 10 times. Next, we used a Docker container developed by Beers et al. (62) to transform the network into an undirected graph. Edges link accounts that were retweeted at least 10 times by at least two of the same users. The resulting graph therefore filters users to those that received some amount of repeated engagement from more than one account rather than those that produced a single viral tweet. There are several advantages to using a coengagement approach. Networks where ties are based on who follows or engages with whom directly may be vulnerable to spam and bot activity (i.e. especially high engagement) and underestimate the importance of popular users who do not follow or engage with many other users. Instead, coengagement network structure reflects how a user’s content is received. Finally, since we only consider retweets on tweets containing vaccination keywords, this approach allows us to focus on connections between users that are particular to their role in the conversation about vaccines. We repeated the analysis on coengagement networks constructed using alternative edge criteria (at least five retweets by at least five of the same individuals and at least two retweets by at least 10 of the same individuals) to evaluate the robustness of our findings to coengagmeent network settings (Section S10 and Figs. S23–S39).

We used the Infomap hierarchical clustering algorithm implemented at mapequation.org to detect communities within the coengagement network. Infomap balances the detection of potential substructures (i.e. subcommunities within communities) against concisely describing a random walker’s movements through the network to determine the total number of levels to use (95, 96). The subcommunities detected using Infomap correspond well to communities detected using the Louvain method, another community detection algorithm (Section S5, Figs. S7 and S8, and Table S4). Coengagement networks were visualized using the open-source software package Gephi with the ForceAtlas2 layout algorithm (97, 98).

### Tagging profiles

We first noted whether a profile indicated that the user primarily tweeted in a language other than English. Because we were unable to assess non-English expertise signals and tweets associated with these users, who may also have reached a substantially different audience compared to English-language accounts, we excluded non-English accounts from our analysis. We also noted accounts that appeared not to represent individuals (e.g. accounts for media groups, nonprofit organizations, governmental agencies, and bot accounts). Non-English and nonindividual accounts (2,549 accounts in total) remained in the network for visualizations and calculations related to network centrality but were excluded from analyses focused on comparing individuals with and without signals of expertise in their profiles.

For the remaining individual profiles, we noted whether there were signals of expertise in the account username or display name (which are listed with tweets in a user’s feed) or the account description (which is only visible if a user mouses over the author’s tweet or directly visits the account) (Fig. S2). Signals of expertise included academic prefixes (e.g. Dr or Professor), suffixes (e.g. MD, MPH, RN, PhD), and professional information (e.g. scientist, retired nurse). We limited our definition of perceived expertise to include training or professional experience in a potentially relevant field but excluded individuals who expressed an interest in a related topic without providing qualifications (e.g. “virology is the coolest”). We included anonymous accounts and ones that may have been parodies (e.g. “Dr Evil” and “The Mad Scientist”) since we expect that users evaluate profiles based on heuristics and without investigating the veracity of information provided (26). We assumed that medical and wellness professionals, including practitioners of alternative medicine, may broadly be perceived as experts regardless of specialty (28). To focus on biomedical expertise, we did not code users from other fields as perceived experts (e.g. science journalists, disability rights advocates, and governmental officials without biomedical backgrounds), acknowledging that these sources may provide trusted and knowledgeable perspectives relevant to health decision-making.

Users that indicated expertise in any part of their profile (names, description, or both) at any point in time were tagged as perceived experts based on experimental evidence that users with biomedical expertise signals in their profiles are perceived to have greater expertise on COVID-19 vaccines (31). Users who may be perceived as biomedical experts based on their name who clarify that they are not biomedical experts in their description (e.g. name: Dr Henry Jekyll, description: PhD in 19th century literature) were still included as perceived experts because users viewing their tweets in the main feed display would not see the description and may assume that they have a medical doctorate or doctoral degree in a biomedical field (as observed in Ref. (31)). This classification underscores the difference between *perceived experts* who may or may not intend to be seen as biomedical experts but are nevertheless viewed as such due to platform design and *self-proclaimed experts* who deliberately claim expertise. Users that changed their profiles over the course of the study in a manner that changed whether they might be perceived as an expert were excluded from the following analyses. Additional examples of perceived expert and perceived nonexpert profiles based on those observed in this coengagement network are provided in Ref. (31). One author tagged all accounts in the coengagement network and two authors tagged a sample of 500 accounts to assess interrater reliability.

### Determining community stance

To understand the stance of individuals in the two largest communities, we analyzed 392 popular tweets by central individuals in both communities (Methods S1.3). For each tweet, three coders assessed stance toward COVID-19 vaccines as positive, negative, or neutral (Methods 1.4). Negative stances toward COVID-19 vaccines were prevalent in tweets from one community, which we refer to as the antivaccine community (Fig. S3). The other community, in which tweets mainly expressed positive stances, was the provaccine community that served as a basis of comparison.

### Academic and low-quality link-sharing

We expanded shortened URLs in tweets by users in the antivaccine and provaccine communities using the RCurl package in R (99). In some cases, this process timed out or links connected to other content shared on Twitter; such links were excluded from the following analysis. Across community and perceived expertise, there was minimal risk of differences in link-sharing frequency leading to bias in the following analyses (Fig. S4). We checked the remaining 79,942 links, first determining whether the domain name was rated “low” or “very-low” quality by Media Bias/Fact Check according to the Iffy Index of Unreliable Sources (100, 101), a common proxy for misinformation sharing (18, 55). To assess sharing of academic research, we checked links to research publications (102) and preprint servers used in biomedical and medical sciences (103). For perceived experts and perceived nonexperts in the two large communities, we calculated: (i) the proportion of assessed links from low-quality or academic research sources and (ii) the proportion of total users that shared at least one link from a low-quality or academic research source in their original posts during April 2021. For each metric, we calculated the binomial proportion 95% CI as $\hat{\;p}\pm 1.96\times \sqrt{\frac{\hat{\;p}(1-\hat{\;p})}{n}}$, where $\hat{\;p}$ is the observed proportion and *n* is the total links or users in a given category. To compare proportions for different categories of users, we conducted two proportion one-tailed *Z*-tests with Yates’ continuity correction to account for the small number of links from low-quality sources shared by the provaccine community. Next, we compared sharing of news sources with partisan biases (right-wing or left-wing), as determined by Media Bias/Fact Check using similar methods (101).

### Network centrality and bridging metrics

We calculated degree, betweenness, and Pagerank centrality to describe network position using the igraph package in R (104). Degree centrality is the number of edges a user has linking it to other users, and users with greater degree centrality share an audience with many other users in the coengagement network (i.e. were retweeted several times by users who also repeatedly retweeted other users). Betweenness centrality is the number of times a given user appears along the shortest path between two other users in the network, meaning that it selects for users who share audiences with sets of users who otherwise do not have much overlap in the people who retweet them. PageRank centrality recursively assigns users a value based on whether they have many connections to other users with high PageRank centrality, selecting users who share audiences with many users whose audiences overlap with those of many others in the coengagement network. We additionally detected users with audiences spanning the antivaccine and provaccine communities using a community bridging score calculated as the minimum number of edges that a user has linking it to either of the two communities. A description of centrality metrics and their interpretation in the coengagement context is provided in Table S1.

We tested whether perceived experts were overrepresented within the group of users ranked highly by each metric by calculating the proportion of perceived experts in the top *n* users ranked by a given metric (where *n* varies between 500 and 50 for all metrics and 10 for bridging score). Several perceived experts and perceived nonexperts had the same bridging scores, leading to ties for ranking in the top 500 and 50 bridges. In these cases, we randomly drew 1,000 samples from the tied users without replacement to complete the set of top bridges and calculated the mean proportion of perceived experts across all samples. To calculate the 95% CI given ties, we subtracted and added the margin of error described above to the 0.025th and 97.5th percentile proportion values across the 1,000 samples, respectively. For the remaining metrics and subsets, we calculated the binomial proportion 95% CI as in the previous section except when examining the 10 top bridges, in which case we calculated the Wilson score interval to account for the small sample size.

To test whether perceived experts were significantly overrepresented in each sample of highly ranked individuals, we conducted two proportion Z-tests with the alternative hypothesis that perceived experts were a significantly greater proportion of the top *n* individuals than individuals in the complement (i.e. individuals who were not among the top *n* individuals ranked by a given metric). When considering the top 10 bridges, we instead used a Fischer’s exact t test to account for the small sample size.

### Matching to assess relative influence of perceived experts

This portion of the analysis was pre-registered on OSF Registries prior to hypothesis testing (https://osf.io/6u3rn). We assessed how perceived expertise affected influence using propensity score matching, a technique to select a sample of perceived nonexperts with a similar covariate distribution to perceived exerts in the dataset so that the average influence of perceived experts and perceived nonexperts may be compared while adjusting for potential confounders (105) (Figs. S17 and S18). Propensity score matching was performed using the MatchIt package in R (106).

We matched on the following covariates: natural logged follower count at the beginning of April 2021, natural logged count of on-topic posts during the study period (i.e. posts included in our data set because they contained a COVID-19 vaccine keyword), account creation date, whether the account was verified, percent of on-topic posts containing links, percent of on-topic tweets that were retweets (vs. original posts), posting time of day, uniformity of posting date across the study period, and subcommunity assignment (Table S2, see Fig. S17 for frequency distributions of matching covariates). We used logistic regression to compute propensity score as the predicted probability that each user is a perceived expert given their covariates. Based on propensity scores, each perceived expert was matched with replacement to their three nearest neighbor perceived nonexperts. To test the first two hypotheses about influence within the antivaccine and provaccine communities, respectively, we included only users in either community. To test the third hypothesis comparing the influence of perceived experts in the antivaccine vs. provaccine community, we calculated the interaction of perceived expertise and community. Sample sizes for all analyses are provided in Table S5. The outcome variables for influence were: PageRank centrality, degree centrality, betweenness centrality, median likes across posts, median retweets across posts, h-index of likes across posts, and h-index of retweets across posts (Table S3). The h-index is the maximum number *h* such that the user wrote at least *h* tweets (including replies and quote tweets) that received at least *h* likes or retweets. We calculated engagements (likes and retweets) for each tweet as the maximum number observed for that tweet in our collection within 28 days of when it was posted. Importantly, we only retrieved like counts for a given tweet when another user engaged with the tweet through a quote tweet or retweet.

We then estimated the effect of perceived expertise on influence by calculating the ATT, which corresponds to the difference in expected influence ((Y(T)) with and without perceived expertise given that a user is a perceived expert (T=1) with covariates drawn from the distributions for the matched users (*X*):


ATT=E[Y(1)−Y(0)∣T=1,X].

Since the engagement metrics were overdispersed counts (median likes, median retweets, h-index likes, and h-index retweets), we used a quasipoisson regression to estimate ATT as the logged risk ratio of engagements for perceived experts compared to perceived nonexperts. ATT was estimated by g-computation using the marginal effects package (107). Using the delta method, cluster-robust standard error was calculated for each metric, with clusters corresponding to sets of perceived nonexperts and perceived experts who were matched to each other.

We report standardized ATT in Fig. 5, calculated by dividing the mean difference for a given metric by the cluster-robust standard error, and analytic 95% CIs. We additionally tested the robustness of our results to different matching parameters and covariates (Figs. S20–S22).

## Supplementary Material

pgae007_Supplementary_Data

## Data Availability

Code and data used to conduct main analyses and reproduce figures are available on Github at: https://github.com/mjharris95/perceived-experts. As explained in the repository, users were anonymized throughout the analysis and in the dataset. Additional information may be provided by the corresponding author on reasonable request.

## Preprints

A preprint of this article is published at https://doi.org/10.1101/2023.07.12.23292568.
