# Supplementary material for: Perceived experts are prevalent and influential within an antivaccine community on Twitter
Source: PNAS Nexus. 2024 Feb 7;3(2):pgae007. doi: 10.1093/pnasnexus/pgae007 (PMC10847722; doi:10.1093/pnasnexus/pgae007)
Supplement: pgae007_Supplementary_Data [file pgae007_supplementary_data.pdf]

Supplementary files: Perceived experts are prevalent and influential within an anti-vaccine community on Twitter

January 4, 2024

Contents

1 Supplementary Methods **2**
1.1 Context on study period (April 2021) 2
1.2 Anonymizing data 2
1.3 Sampling popular tweets to determine stance 2
1.4 Classifying tweet stance 3

2 Coengagement network visualization without annotations **4**

3 Variable definitions **5**

4 Tweet and user characteristics **8**

5 Subcommunity descriptions and visualization **13**

6 Network visualization with nodes scaled by different metrics **18**

7 Main matching analyses **22**

8 Distribution of matching covariates and outcomes **28**

9 Sensitivity to matching specifications **34**

10 Sensitivity to coengagement network parameters **41**

1 Supplementary Methods

This section expands on the methods provided in the main text.

1.1 Context on study period (April 2021)

We constrained our analysis to April 2021, noting that all individuals sixteen and older were eligible for vaccination by April 19th, marking this time period as an especially critical window for vaccine decision-making [Roy, 2021]. Further, administration of the Johnson & Johnson (Janssen) vaccine was paused in the United States between April 13th and April 23rd while the Centers for Disease Control and Prevention (CDC) and Food and Drug Administration (FDA) investigated a safety signal involving six reported cases of severe blood clots [U.S. Food and Drug Administration, 2021]. Focusing on April 2021 also allows us to examine how different communities reacted to credible news of a serious but rare vaccine safety signal. During the same time period, fact checkers and researchers responded to several false claims about vaccination, including rumors that vaccinated people were able to "shed" vaccine components from COVID-19 vaccines that might infect and harm unvaccinated people [The Virality Project, 2021].

1.2 Anonymizing data

Individual users are not named in the analyses, reinforcing that we aim to characterize the *group* of perceived experts within the anti-vaccine community instead of focusing on individual, high-profile examples. User profiles and numeric identifiers for accounts used by Twitter were stored separately from tweets, which were instead linked to the anonymous identifiers we assigned. We also stored a dictionary that allows for translation between these two distinct numeric identifiers and utilized the dictionary to connect statistics about tweets to properties of the network and users (e.g., to determine community stance after tagging the stance of a sample of popular tweets, to compare the types of links shared depending on perceived expertise and community, and to collect matching covariates based on Twitter activity and engagements). In tweets we coded for stance, we anonymized mentions of other usernames and removed links to other posts and images on Twitter to prevent user identification. Therefore, researchers were blinded to user identity when assessing tweet stance.

1.3 Sampling popular tweets to determine stance

Of the 251,040 tweets that received retweets during April 2021, we tagged a subset posted by the ten perceived experts and perceived non-experts in both communities with the greatest degree centrality. We collected the ten most retweeted tweets by the forty selected users. A few of the users had fewer than ten tweets that were retweeted during the study period, so we instead retrieved

all of their tweets that received retweets in April. In total, we reviewed 392 tweets (with three coders assessing each tweet).

1.4 Classifying tweet stance

Stance was evaluated as positive if the tweet supported vaccination, including by providing evidence that COVID-19 vaccines are safe and effective. Tweets expressing the opposite view were coded as negative. Stance was often implicit. For example, calls for deployment of vaccines to "hotspots" with elevated disease burden suggest that the author believes the vaccine can prevent disease, even if this rationale is not stated directly. Unless the author refuted or negatively reacted to a quote they provided, a tweet they quote tweeted, or an article they linked, the post was assumed to take the same stance as referenced sources. Tweets containing multiple contrasting viewpoints or providing information without a clearly implied position (e.g., sharing statistics about the pace of vaccination without any additional commentary) were coded as neutral. The neutral tag was also applied to tweets if the coder was uncertain about the argument being made or context was missing (e.g., criticism of an article that could not be retrieved). We noted many tweets during this time period that opposed vaccine mandates and coded these tweets as neutral unless the author justified their position using a specific argument regarding vaccine safety or efficacy. Tweets comparing multiple types of vaccines with negative stances toward some and positive stances toward others were tagged neutral. Tweets that took a uniform stance toward different types of COVID-19 vaccines while explaining their differences were coded based on the corresponding stance.

2 Coengagement network visualization without annotations

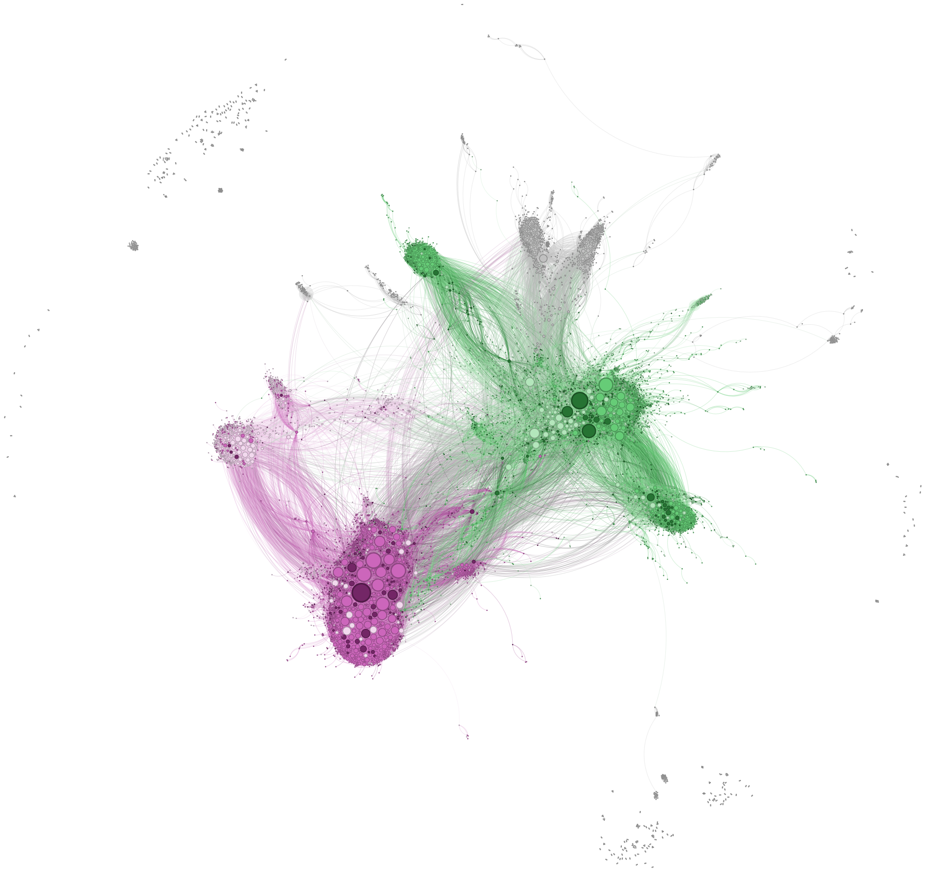

Figure 1: **The coengagement network of users tweeting about COVID-19 vaccines is divided into two large communities.** Users are represented as circles and scaled by degree centrality. Edges connect users that were retweeted at least ten times by at least two of the same users. Nodes in the two largest communities detected using the Infomap algorithm are colored in pink (anti-vaccine) and green (pro-vaccine). Shades indicate account type: non-individual and non-English accounts excluded from analyses (light); perceived non-expert (medium); and perceived experts (dark). Nodes outside of the two largest communities are gray. Each edge is colored based on the color of one of the two nodes it connects, randomly selected. Note that this figure is identical to Main Text Figure 1 without annotations and in higher resolution.

3 Variable definitions

This section provides explanations of variables used in our analyses, including centrality metrics (Supplemental Table 1) and matching covariates (Supplemental Table 2) and influence metrics used in propensity score matching (Supplemental Table 2).

Metric name	Description	Coengagement interpretation
Degree centrality	Number of edges connecting a given node to other nodes	Selects users who share an audience with many other users; proxy for audience size
Betweenness centrality	Number of times a given node appears along the shortest path between two other nodes in the network	Selects users who share audience with users that otherwise do not have much overlap in their audiences
PageRank centrality	Recursively assigns nodes a value based on whether they are connected to other nodes with high PageRank centrality	Selects users who share audiences with many other users whose audiences overlap with those of many others in the coengagement network
Community bridging	Takes the minimum of the number of edges that a node has connecting it to nodes in any focal communities	Selects users who share an audience with relatively many users in both the anti- and pro-vaccine communities

Table 1: Description of different centrality metrics and their interpretation in the context of coengagement networks.

**Matching covariates**	**Description**
Creation date	Continuous variable. Date and time that account was created.
Follower count (log)	Continuous variable. Natural log of the earliest follower count retrieved for a user within the dataset.
Account verified?	Binary variable. Whether account was verified at any point in April of 2021. Note: twelve users in the dataset, eight of whom were in the two largest communities, became verified during the study period.
On-topic post count (log)	Continuous variable. Natural log of the total number of posts (including quote tweets and replies) and retweets by a user in April containing COVID-19 vaccine keywords.
Percent retweets	Continuous variable. Percent of user's on-topic posts in April that were retweets.
Percent with links	Continuous variable. Percent of user's on-topic posts in April that contained links.
Morning poster?	Binary variable. Whether at least one third of a user's on-topic posts were between 6 AM and noon Eastern Time.
Afternoon poster?	Binary variable. Whether at least one third of a user's on-topic posts were between noon and 6 PM Eastern Time.
Evening poster?	Binary variable. Whether at least one third of a user's on-topic posts were between 6 PM and midnight Eastern Time.
Night poster?	Binary variable. Whether at least one third of a user's on-topic posts were between midnight and 6 AM Eastern Time.
Uniform post dates?	Binary variable. Whether the dates of a user's on-topic post were uniformly spaced across the study period. Calculated by conducting a chi-squared test comparing the distribution of post dates to a uniform distribution and testing whether the resulting p-value is less than 0.10.
Subcommunity 1-2	Binary variable. Whether the user is in subcommunity 1-2 (the largest anti-vaccine subcommunity).
Subcommunity 1-1	Binary variable. Whether the user is in subcommunity 1-1 (the second-largest anti-vaccine subcommunity).
Subcommunity 2-1	Binary variable. Whether the user is in subcommunity 2-1 (the largest pro-vaccine subcommunity).
Subcommunity 2-2	Binary variable. Whether the user is in subcommunity 2-2 (the pro-vaccine subcommunity focused on vaccination in Canada).

Table 2: Description of covariates used for matching.

**Outcome variable**	**Description**
Median likes	Count variable. Median number of likes received on all original (non-retweet) on-topic posts. Reported ATT is the natural log of risk ratio.
h-index likes	Count variable. h-index for likes received across all original (non-retweet) on-topic posts. Reported ATT is the natural log of risk ratio.
Median retweets	Count variable. Median number of retweets received on all original (non-retweet) on-topic posts. Reported ATT is the natural log of risk ratio.
h-index retweets	Count variable. h-index for retweets received across all original (non-retweet) on-topic posts. Reported ATT is the natural log of risk ratio.
Degree centrality	Continuous variable. Degree centrality within coengagement network.
PageRank centrality	Continuous variable. PageRank centrality within coengagement network.
Betweeness centrality	Continuous variable. Betweenness centrality within coengagement network.

Table 3: Description of influence metrics that were outcomes for matching.

4 Tweet and user characteristics

This section contains additional information about expertise signals provided by users (Supplemental Figure 2), the vaccine stances of popular tweets in the the anti- and pro-vaccine communities (Supplemental Figure 3), link-sharing behavior for users depending on perceived expertise and community (Supplemental Figure 4), the overlap in users who shared both academic and low quality sources (Supplemental Figure 5), and partisan link-sharing by users depending on community and perceived expertise (Supplemental Figure 6).

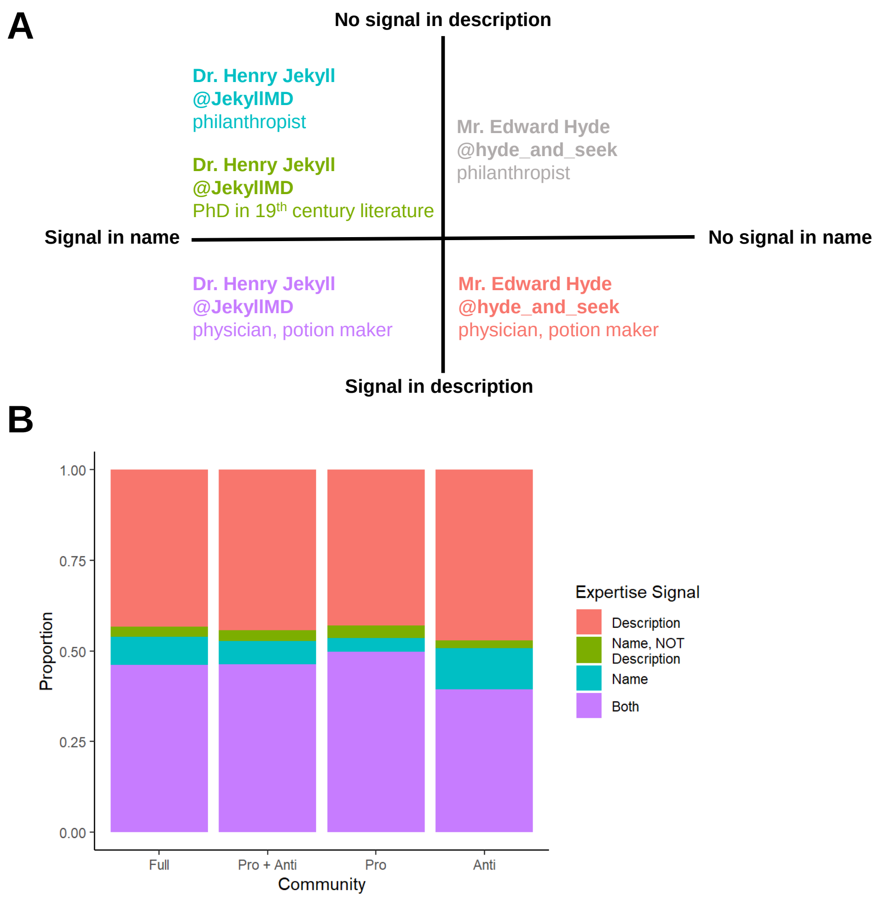

Figure 2: **Signals of expertise may appear in different parts of the profile.** Panel A (top) gives examples of different ways that expertise signals may be presented. Starting in the bottom right and moving counterclockwise: signal in description only (red), signal in name and description (purple), signal in name but description clarifies user is not an expert (green), or signal in name only no information about perceived expertise in description (blue). The gray profile has no signals of expertise and is therefore not a perceived expert. Panel B (bottom) is the proportion of perceived experts that provide expertise signals in each way across different group (x-axis): the full coengagement network, the pro- and anti-vaccine communities combined, the pro-vaccine community alone, and the anti-vaccine community alone.

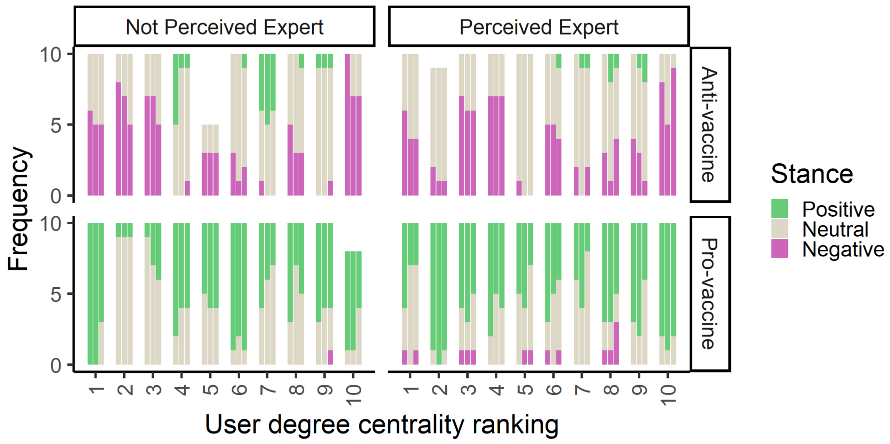

Figure 3: **User stance is largely consistent within the two largest communities.** Plots display tweet stance across the ten perceived non-experts (left column) and ten perceived experts with the greatest degree centrality (right column) in the anti- and pro-vaccine communities (top and bottom row respectively). Users are ranked and arranged by degree centrality (x-axis). Three coders assessed the stance of the most highly retweeted tweets from each user (up to ten tweets per user), and results from all coders are displayed side-by-side for each user. Bars are shaded by the number of tweets assessed as positive (green), neutral (gray), or negative (pink).

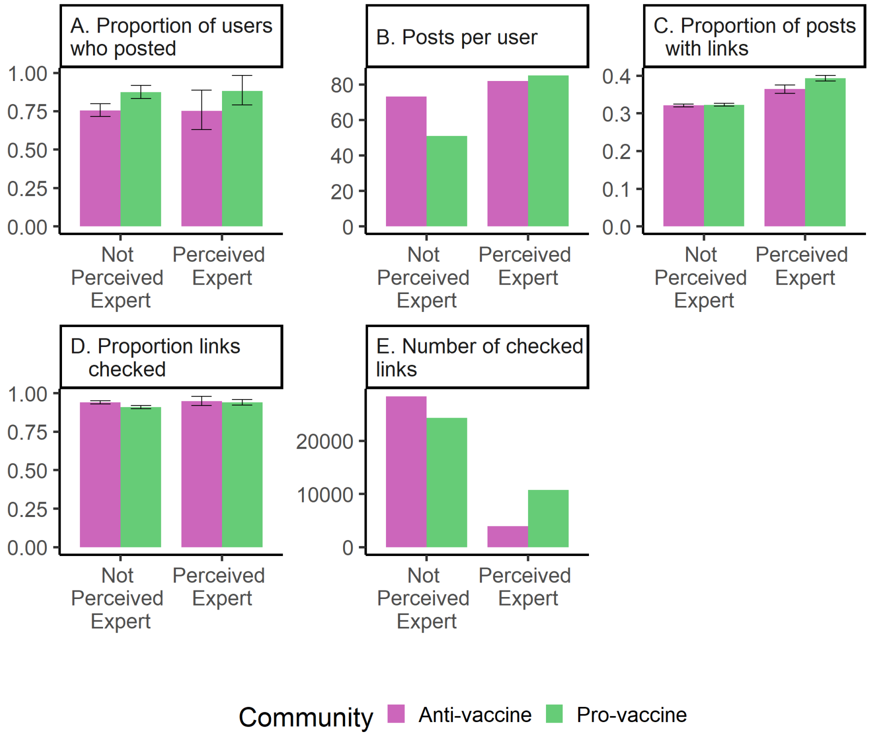

Figure 4: **Link-sharing activity by community and perceived expertise.** Each panel compares a different metric of link-sharing by perceived experts and perceived non-experts in the anti- (pink) and pro- (green) vaccine communities. The metrics are: (A) proportion of users who posted original content in April (B) average number of posts per user (C) proportion of all tweets with links (D) proportion of links that were checked (did not link back to Twitter or time out during link expansion) (E) the total number of checked links in a given category. Error bars give 95% binomial proportion confidence intervals for proportions in panel A, C, and D.

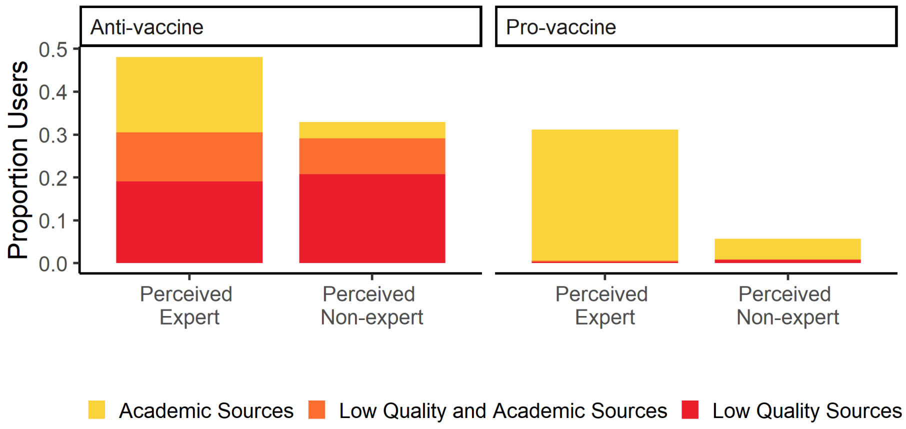

Figure 5: **There is modest overlap between individuals who share low quality and academic sources in the anti-vaccine community.** Each bar shows the proportion of users in a given category who shared at least one low quality source and no academic sources (red), at least one academic source and no low quality sources (yellow), or at least one academic source and at least one low quality source (orange), Proportions are broken down by perceived experts and perceived non-experts (x-axis) in the anti-vaccine (left panel) and pro-vaccine (right panel) communities.

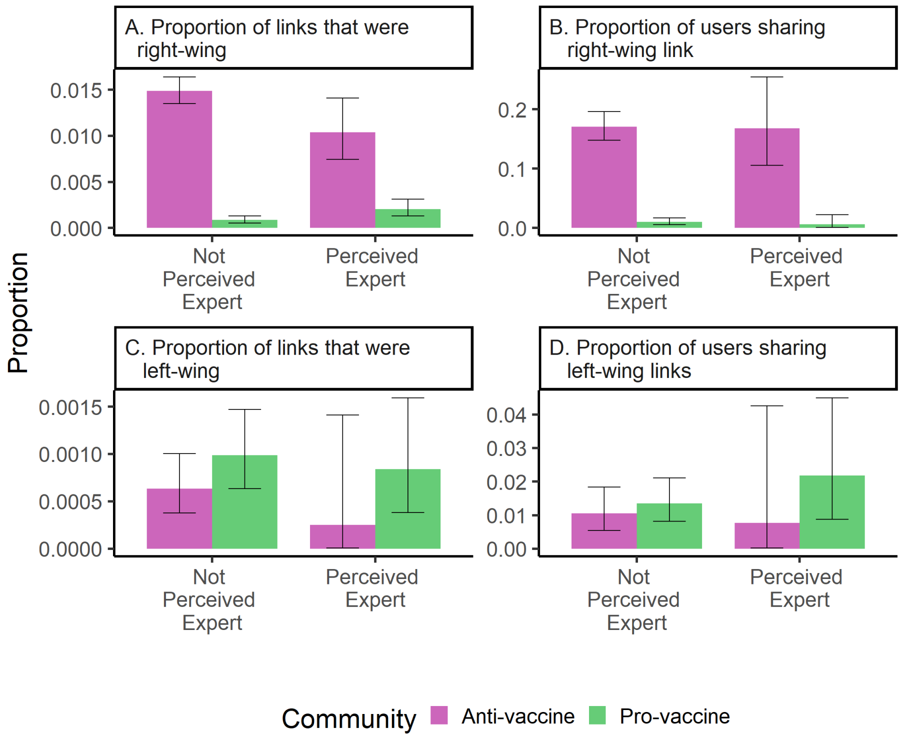

Figure 6: **Partisan link-sharing activity across communities and perceived expertise.** Each panel compares a different metric of link-sharing by perceived experts and perceived non-experts in the anti- (pink) and pro- (green) vaccine communities. The metrics are: (A) proportion of checked links that were from right-wing sources, (B) proportion of users that shared at least one right-wing source, (C) proportion of checked links that were from left-wing sources, and (D) proportion of users that shared at least one left-wing source. 95% binomial proportion confidence intervals are indicated by black error bars.

5 Subcommunity descriptions and visualization

Infomap is a hierarchical community detection algorithm, allowing the detection of subcommunities [Holmgren et al., 2022]. In this section, we visualize subcommunities detected using Infomap (Supplemental Figure 7) and note that these subcommunities closely resemble communities detected using the Louvain method, suggesting that our results are robust to choice of community detection algorithm. We also provide a table of subcommunity properties (Supplemental Table 4).

We reviewed a sample of popular tweets from each subcommunity to label

subcommunities based on topic. Within the pro-vaccine community, the four most popular subcommunities included: the largest (main) subcommunity that generally discussed vaccine safety and efficacy and focused on vaccination in the United States, a subcommunity of predominantly non-individual accounts that posted news stories with headlines, a subcommunity focused on vaccination in Canada, and a subcommunity focused on vaccination in Australia. Tweets in the latter two subcommunities often criticized the pace of vaccination as too slow, blaming specific politicians. The four most popular anti-vaccine subcommunities included two large (main) subcommunities that we labeled as main anti (A) and main anti (B). The distinction between the two subcommunities may be related to main anti (A)'s greater connection to followback clusters, or densely connected groups of accounts that frequently retweet each other and a small set of accounts outside of their group [Beers et al., 2023], although we did not test this hypothesis directly. Again, these subcommunities generally discussed vaccine safety and efficacy, with some emphasis on vaccination in the United States. The two next largest subcommunities in the anti-vaccine community consisted largely of non-English accounts with profiles in French or Italian. The third largest community that was excluded from the main analysis tweeted about vaccination in India ([Hagen et al., 2022, Boucher et al., 2021]), which broke into two subcommunities: India (A) and India (B). It was not immediately clear whether there were any differences in the content posted by users in either subcommunity.

The communities detected using the Louvain method closely resembled these subcommunities, although the media subcommunity in the pro-vaccine community was combined with the main pro-vaccine subcommunity. Across subcommunities, all users were fully contained within their equivalent Louvain subcommunity except in the case of anti (A), as some users in the boundary between anti (A) and anti (B) were classified into the latter subcommunity. There were 2004 users in the largest Louvain communities that were not in any of the largest Infomap subcommunities, meaning that the Louvain communities were generally more expansive.

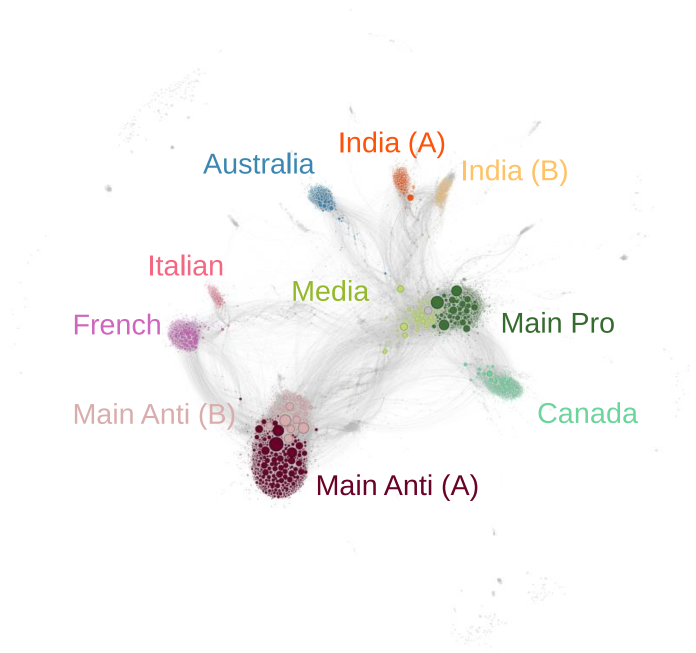

Figure 7: **The main subcommunities of the coengagement network of users tweeting about COVID-19 vaccines.** Users are represented as circles and scaled by degree centrality. Edges connect users that were retweeted at least ten times by at least two of the same users. All edges are gray. Nodes in the ten largest subcommunities detected using the Infomap algorithm are colored in different shades of pink and red if they are in the anti-vaccine community (top to bottom: Italian, French, Main Anti (B), Main Anti (A)), green and blue if they are in the pro-vaccine community (top to bottom: Australia, Media, Main Pro, and Canada), and shades of orange if they are in the community focused on vaccination in India (top to bottom: India (A), India (B)). Nodes outside of the ten largest subcommunities are gray.

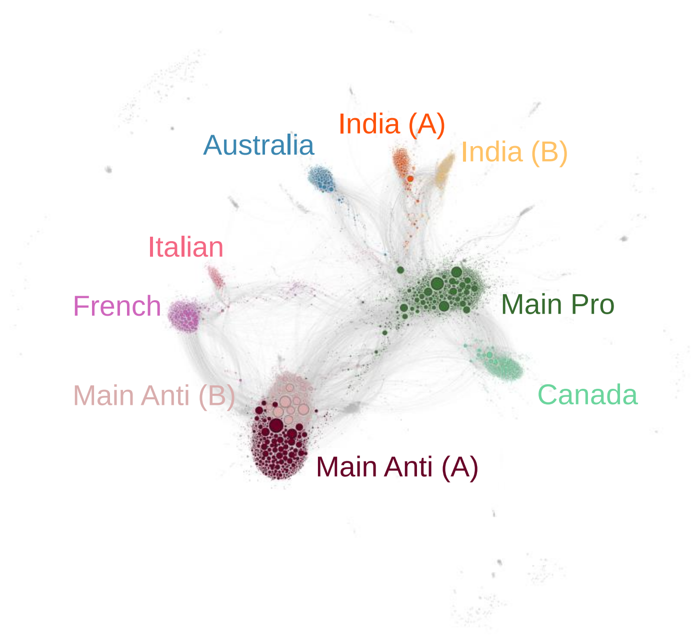

Figure 8: **The main communities of the coengagement network of users tweeting about COVID-19 vaccines detected using the Louvain algorithm roughly correspond to subcommunities detected using the Infomap algorithm (compare to Supplemental Figure 7).** Users are represented as circles and scaled by degree centrality. Edges connect users that were retweeted at least ten times by at least two of the same users. All edges are gray. Nodes in the nine largest communities detected using the Louvain algorithm are colored in different shades of pink if they are in the anti-vaccine community (top to bottom: Italian, French, Main Anti (B), Main Anti (A)), green if they are in the pro-vaccine community (top to bottom: Australia, Main Pro, and Canada), and shades of orange if they are in the community focused on vaccination in India (top to bottom: India (A), India (B)). Nodes outside of the nine largest subcommunities are gray.

Subcommunity Code	Name	Louvain Equivalent	Community	Total users	Included users	Perceived Experts	Count in Louvain
1 1	Anti (A)	Anti (A)	Anti	744 (0.28)	684 (0.92)	76 (0.11)	662 (0.89)
2 1	Main Pro	Main Pro	Pro	737 (0.21)	579 (0.79)	84 (0.15)	737 (1)
1 2	Anti (B)	Anti (B)	Anti	538 (0.20)	491 (0.91)	40 (0.08)	538 (1)
1 3	French	French	Anti	425 (0.16)	90 (0.21)	7 (0.08)	425 (1)
2 2	Canada	Canada	Pro	385 (0.11)	283 (0.74)	56 (0.20)	385 (1)
2 4	Australia	Australia	Pro	316 (0.09)	289 (0.91)	42 (0.15)	316 (1)
2 3	Media	Main Pro	Pro	258 (0.07)	49 (0.19)	8 (0.16)	258 (1)
3 2	India (A)	India (A)	India	239 (0.35)	194 (0.81)	17 (0.09)	239 (1)
3 1	India (B)	India (B)	India	186 (0.27)	162 (0.87)	17 (0.10)	186 (1)
1 5	Italian	Italian	Anti	183 (0.07)	50 (0.27)	2 (0.04)	183 (1)

Table 4: **Properties of subcommunities detected using the Infomap community detection algorithm (displayed in Supplemental Figure 7).** From left to right, the columns are as follows: the code for the subcommunity (where the first digit corresponds to the community code and the second digit corresponds to the subcommunity code); a subcommunity name determined by reviewing a sample of popular tweets; the name of the equivalent subcommunity detected using the Louvain community detection algorithm; the name of the community to which the subcommunity belongs; the total number of accounts in the subcommunity and, in parentheses, the proportion of total users in the community that are part of a given subcommunity; the number of users meeting the criteria for inclusion (i.e., English profile, individual, unchanged perceived expertise) and, in parentheses, the proportion of users in the subcommunity meeting the criteria for inclusion in analyses; the number of perceived experts in the subcommunity and, in parentheses, the proportion of users in the subcommunity included in the analysis who are perceived experts; and the count of users in a given Infomap subcommunity who are also in the corresponding Louvain community and, in parentheses, the proportion of users in the Infomap subcommunity who are in the corresponding Louvain community (1 indicates a subcommunity is fully contained within the corresponding Louvain community).

6 Network visualization with nodes scaled by different metrics

This section contains visualizations of the coengagement network where nodes are scaled and labeled according to each network metric used in the analyses: community bridging (Supplemental Figure 9), degree centrality (Supplemental Figure 10), betweenness centrality (Supplemental Figure 11), and PageRank centrality (Supplemental Figure 12). These figures allow comparisons of how different nodes are ranked across each metric.

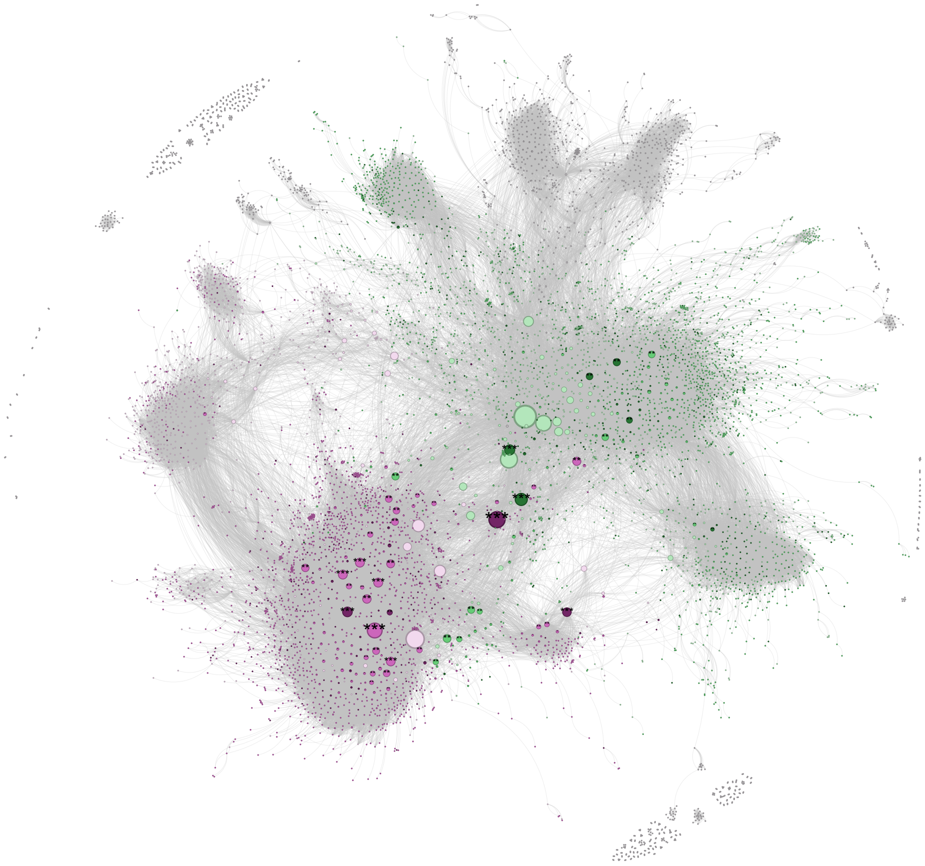

Figure 9: **Node size corresponds to *community bridging* score.** As in Main Text Figure 1, users are represented as circles. Edges connect users that were retweeted at least ten times by at least two of the same users. All edges are gray. Nodes in the two largest communities detected using the Infomap algorithm are colored in pink (anti-vaccine) and green (pro-vaccine). Shades indicate account type: non-individual and non-English accounts excluded from analyses (light); perceived non-expert (medium); and perceived experts (dark). Nodes outside of the two largest communities are gray. Users that rank in the top 500, top 50, and top ten users by community bridging across the whole coengagement network are labeled with one, two, and three stars respectively.

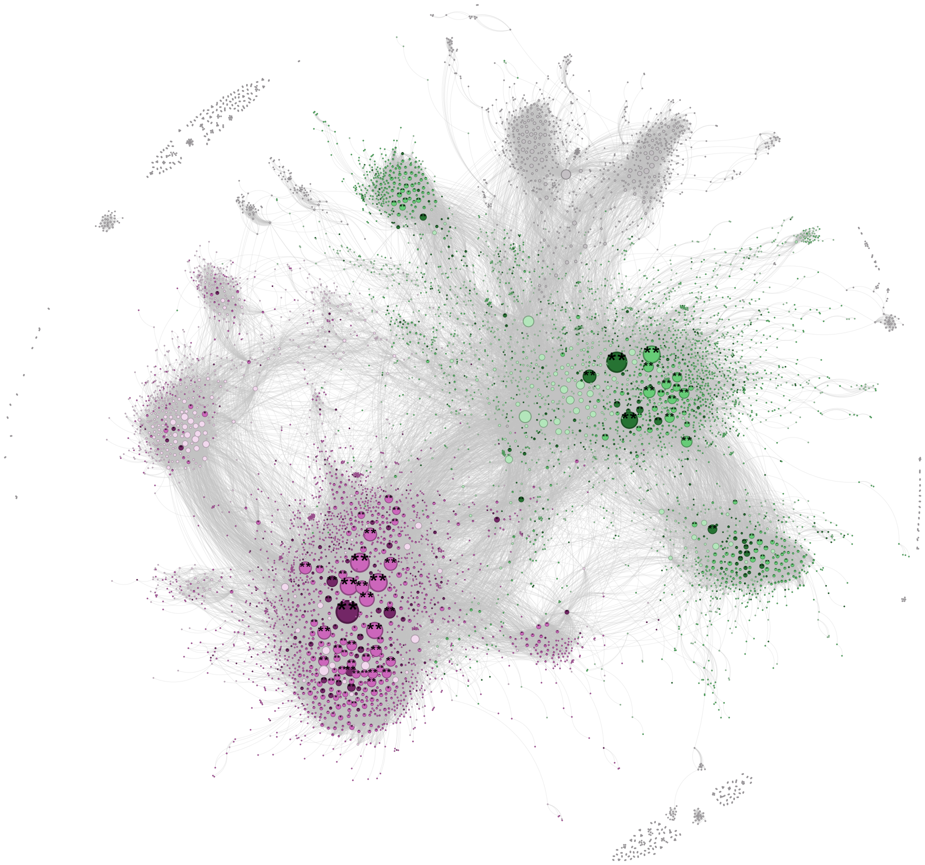

Figure 10: **Node size corresponds to *degree centrality.*** As in Main Text Figure 1, users are represented as circles. Edges connect users that were retweeted at least ten times by at least two of the same users. All edges are gray. Nodes in the two largest communities detected using the Infomap algorithm are colored in pink (anti-vaccine) and green (pro-vaccine). Shades indicate account type: non-individual and non-English accounts excluded from analyses (light); perceived non-expert (medium); and perceived experts (dark). Nodes outside of the two largest communities are gray. Users that rank in the top 500 and top 50 users by degree centrality within the anti-vaccine or pro-vaccine community are labeled with one and and two stars.

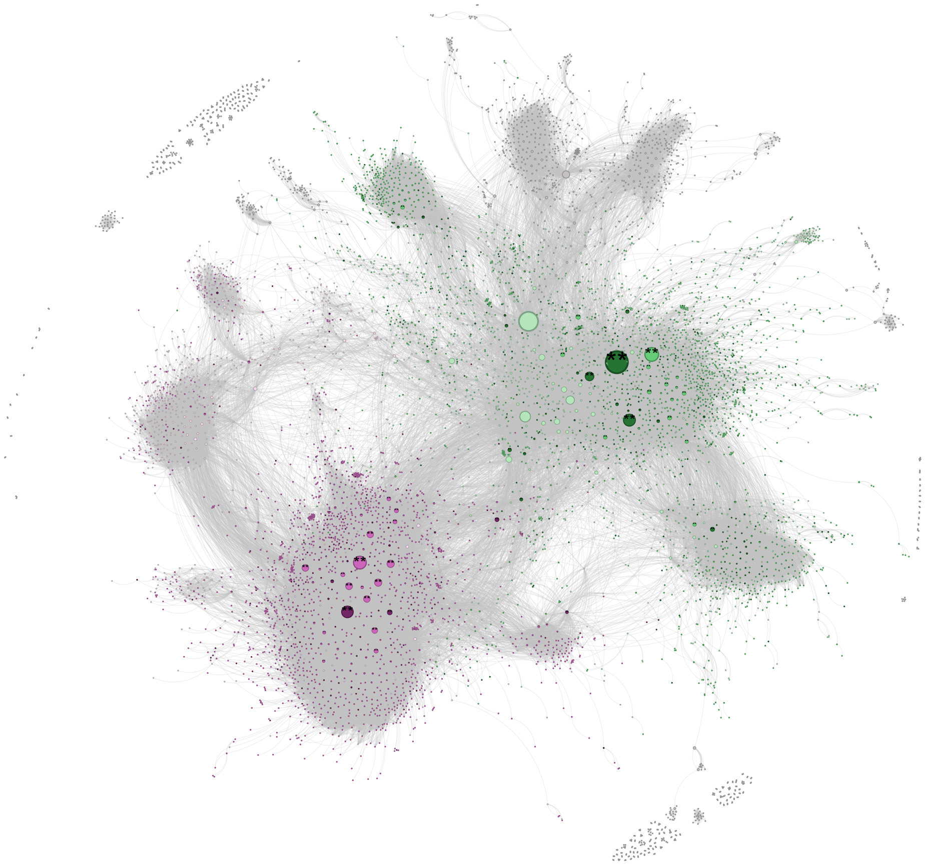

Figure 11: **Node size corresponds to *betweenness centrality*.** As in Main Text Figure 1, users are represented as circles. Edges connect users that were retweeted at least ten times by at least two of the same users. All edges are gray. Nodes in the two largest communities detected using the Infomap algorithm are colored in pink (anti-vaccine) and green (pro-vaccine). Shades indicate account type: non-individual and non-English accounts excluded from analyses (light); perceived non-expert (medium); and perceived experts (dark). Nodes outside of the two largest communities are gray. Users that rank in the top 500 and top 50 users by betweenness centrality within the anti-vaccine or pro-vaccine community are labeled with one and and two stars.

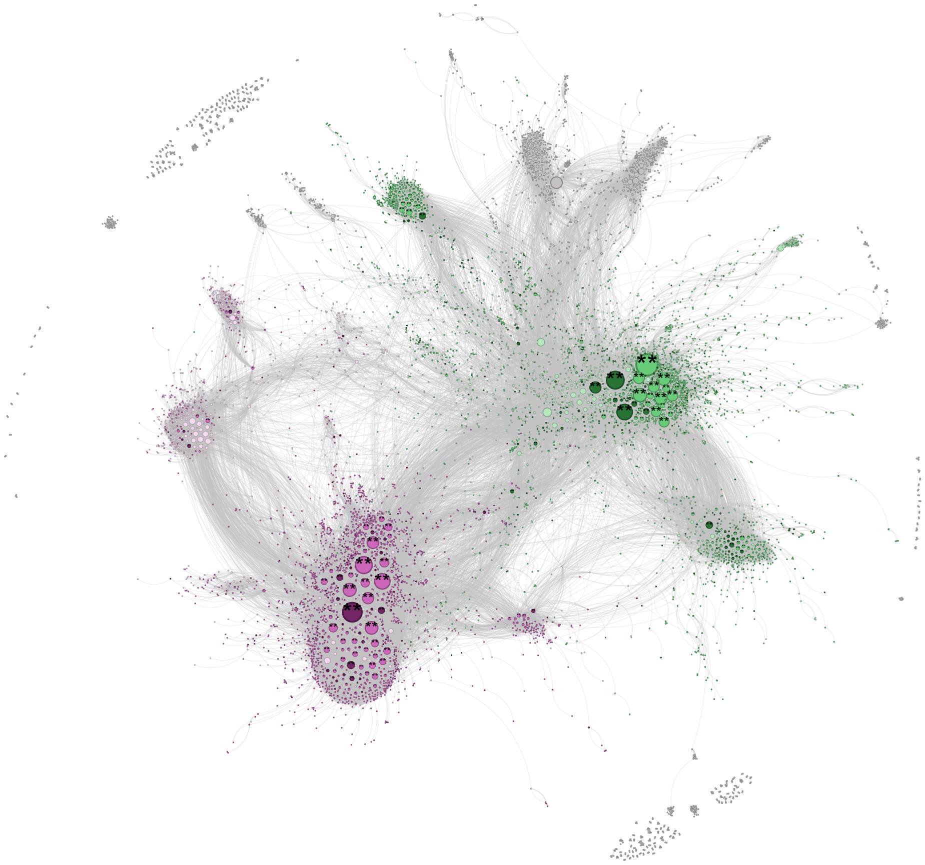

Figure 12: **Node size corresponds to *PageRank centrality.*** As in Main Text Figure 1, users are represented as circles. Edges connect users that were retweeted at least ten times by at least two of the same users. All edges are gray. Nodes in the two largest communities detected using the Infomap algorithm are colored in pink (anti-vaccine) and green (pro-vaccine). Shades indicate account type: non-individual and non-English accounts excluded from analyses (light); perceived non-expert (medium); and perceived experts (dark). Nodes outside of the two largest communities are gray. Users that rank in the top 500 and top 50 users by PageRank centrality within the anti-vaccine or pro-vaccine community are labeled with one and and two stars.

7 Main matching analyses

This section provides sample sizes (Supplemental Table 5), covariate balance before and after matching (Supplemental Figure 13 for H1, Supplemental Figure 14 for H2, Supplemental Figure 15 for H3), and statistics related to estimated

average treatment effect on the treated (ATT) (Supplemental Table 6 for H1, Supplemental Table 7 for H2, and Supplemental Table 8 for H3). The values in Supplemental Table 6 and Supplemental Table 7 are visualized in Main Text Figure 5. We also visualize the difference in the estimated ATT between the anti- and pro-vaccine communities in Supplemental Figure 16, corresponding to Supplemental Table 8.

	Perceived non-experts	Perceived experts
Unmatched (Anti-vaccine)	1652	183
Matched (Anti-vaccine)	549	183
Unmatched (Pro-vaccine)	1819	379
Matched (Pro-vaccine)	1137	379
Unmatched (All users)	3471	562
Matched (All users)	1686	562

Table 5: Sample size of perceived non-experts and perceived experts before and after propensity score matching in the anti-vaccine community (H1), pro-vaccine community (H2), or both (H3).

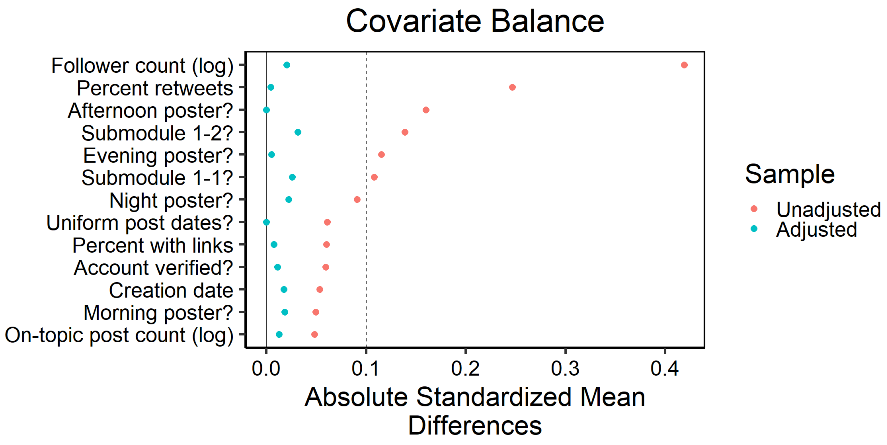

Figure 13: **Love plot demonstrating the balance across matching covariates for individuals in the anti-vaccine community before (orange) and after (blue) propensity score matching was performed to test H1.** Each row corresponds to a different matching covariate (described in Supplemental Table 2. The x-axis is absolute standardized mean difference, where values closer to zero correspond to better balance. The horizontal line indicates 0.1, the threshold below which balance is generally considered good.

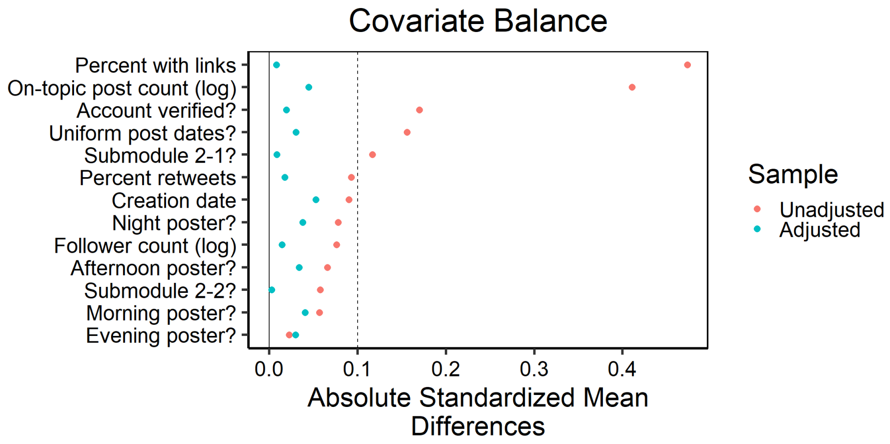

Figure 14: **Love plot demonstrating the balance across matching covariates for individuals in the pro-vaccine community before (orange) and after (blue) propensity score matching was performed to test H2.** Each row corresponds to a different matching covariate (described in Supplemental Table 2. The x-axis is absolute standardized mean difference, where values closer to zero correspond to better balance. The horizontal line indicates 0.1, the threshold below which balance is generally considered good.

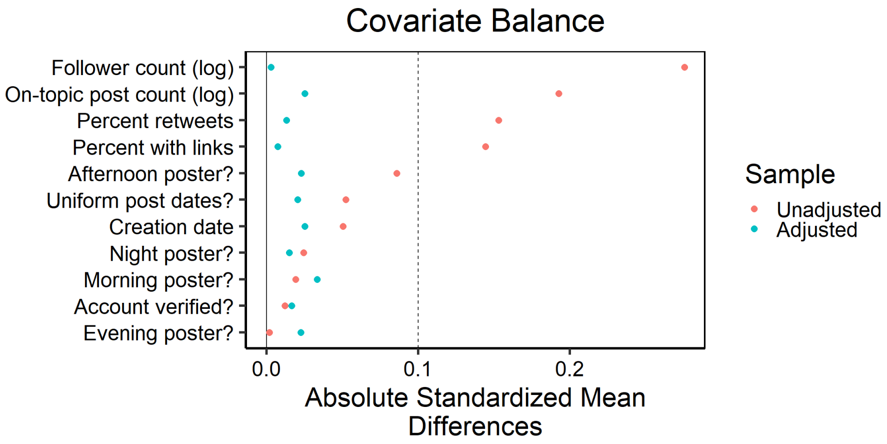

Figure 15: **Love plot demonstrating the balance across matching covariates for individuals in the pro- and anti-vaccine communities before (orange) and after (blue) propensity score matching was performed to test H3.** Each row corresponds to a different matching covariate (described in Supplemental Table 2. The x-axis is absolute standardized mean difference, where values closer to zero correspond to better balance. The horizontal line indicates 0.1, the threshold below which balance is generally considered good.

Outcome	Estimate	p-value	Std. Error
Median Likes (log(RR))	0.04 (-0.27, 0.35)	0.808	0.16
h-index Likes (log(RR))	0.21 (0.12, 0.30)	0.000	0.05
Median Retweets (log(RR))	0.36 (0.02, 0.69)	0.039	0.17
h-index Retweets (log(RR))	0.19 (0.09, 0.28)	0.000	0.05
Degree Centrality	4.26 (-8.84, 17.35)	0.524	6.68
PageRank Centrality	3.22e-05 (-6.89e-05, 1.33e-04)	0.532	0.00
Betweenness Centrality	2796.16 (-11936.24, 17528.56)	0.710	7516.67

Table 6: **Average treatment effect on the treated within the anti-vaccine community (H1).** Each row corresponds to results of the matching analysis for a different outcome variable, named in the first column and described in Supplemental Table 2. The estimate column gives the average treatment effect on the treated with the corresponding 95% confidence interval in parentheses. Positive values indicate greater influence for perceived experts compared to perceived non-experts. The p-value and standard error for each estimate are also provided.

Outcome	Estimate	p-value	Std. Error
Median Likes (log(RR))	0.12 (-0.27, 0.51)	0.540	0.20
h-index Likes (log(RR))	0.25 (0.18, 0.32)	0.000	0.04
Median Retweets (log(RR))	0.23 (-0.07, 0.53)	0.127	0.15
h-index Retweets (log(RR))	0.24 (0.16, 0.31)	0.000	0.04
Degree Centrality	6.43 (0.01, 12.85)	0.050	3.28
PageRank Centrality	5.24e-05 (-7.52e-06, 1.12e-04)	0.087	0.00
Betweenness Centrality	14800.20 (771.39, 28829.00)	0.039	7157.69

Table 7: **Average treatment effect on the treated within the pros-vaccine community (H2).** Each row corresponds to results of the matching analysis for a different outcome variable, named in the first column and described in Supplemental Table 2. The estimate column gives the average treatment effect on the treated with the corresponding 95% confidence interval in parentheses. Positive values indicate greater influence for perceived experts compared to perceived non-experts. The p-value and standard error for each estimate are also provided. For engagement metrics, we compare the natural log of the risk ratio of a given engagement for perceived experts compared to perceived non-experts.

Outcome	Estimate	p-value	Std. Error
Median Likes (log(RR))	3.23e-04 (-5.43e-01, 5.43e-01)	0.999	0.28
h-index Likes (log(RR))	-0.01 (-0.13, 0.11)	0.838	0.06
Median Retweets (log(RR))	0.17 (-0.29, 0.63)	0.469	0.24
h-index Retweets (log(RR))	-0.03 (-0.15, 0.10)	0.685	0.06
Degree Centrality	-1.53 (-16.51, 13.45)	0.841	7.64
PageRank Centrality	-1.82e-05 (-1.36e-04, 9.99e-05)	0.763	0.00
Betweenness Centrality	-12009.55 (-32320.33, 8301.23)	0.246	10362.83

Table 8: **Difference in average treatment effect on the treated between the pro-vaccine and pro-vaccine community (H3).** Each row corresponds to results of the matching analysis for a different outcome variable, named in the first column and described in Supplemental Table 2. The estimate column gives the difference between the average treatment effect on the treated for the anti-vaccine community versus the pro-vaccine community with the corresponding 95% confidence interval in parentheses. Positive values indicate greater a influence boost for perceived experts in the anti-vaccine community compared to the pro-vaccine community. The p-value and standard error for each estimate are also provided.

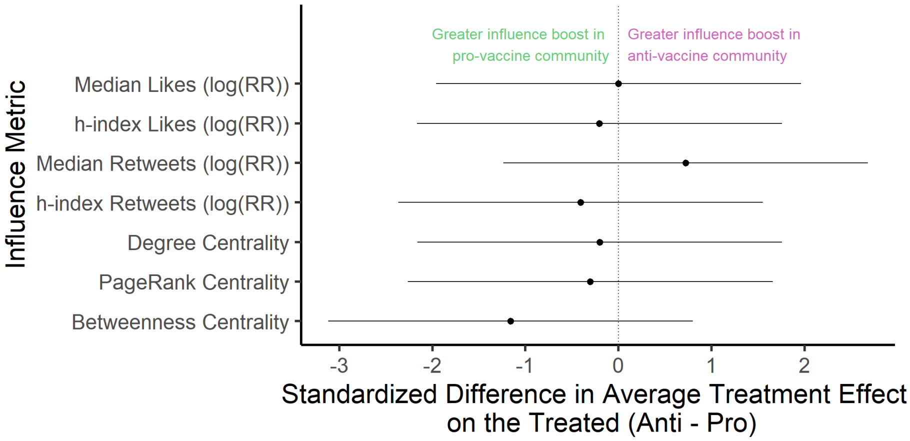

Figure 16: **There is no significant difference in the influence boost for perceived experts between the anti- and pro-vaccine communities**. For each influence metric (y-axis, Supplemental Table 3), we plot the difference in the standardized average treatment effect on the treated (ATT) between the pro- and anti-vaccine communities as a point and corresponding 95% confidence interval (Supplemental Table 8). Positive values (to the right of the vertical line) indicate a greater influence boost for perceived experts in the anti-vaccine commmunity compared to the pro-vaccine community. None of the differences were significant at the $p < 0.05$ level.

8 Distribution of matching covariates and outcomes

In this section, we show the frequency distributions of matching covariates (listed in Supplemental Table 2) and influence metrics (listed in Supplemental Table 3) in the anti-vaccine and pro-vaccine communities across perceived experts, perceived non-experts (full population prior to matching and matched subset). Propensity score matching made covariate distributions more similar between perceived experts and perceived non-experts (Supplemental Figure 17) and generally reduced differences in mean values for outcome metrics between perceived experts and perceived non-experts (Supplemental Figure 18), This finding suggests that perceived expertise may be associated with covariates that also increase influence, justifying the decision to use propensity score matching.

This section also investigates why, despite being overrepresented amongst users with the greatest centrality (Main Text Figure 3), perceived experts do not, on average, have greater centrality than the matched set of perceived non-experts (Main Text Figure 5). Matching generally reduces differences in the frequency distribution for perceived experts versus perceived non-experts across centrality metrics, suggesting that matching covariates may contribute to the greater frequency of perceived experts with high centrality. Balancing therefore may partially reduce the difference in mean centrality between perceived experts and perceived non-experts. However, perceived experts generally remain overrepresented in the right tail of the distribution after matching, even while this effect does not lead to a significant increase in the mean centrality of perceived experts.

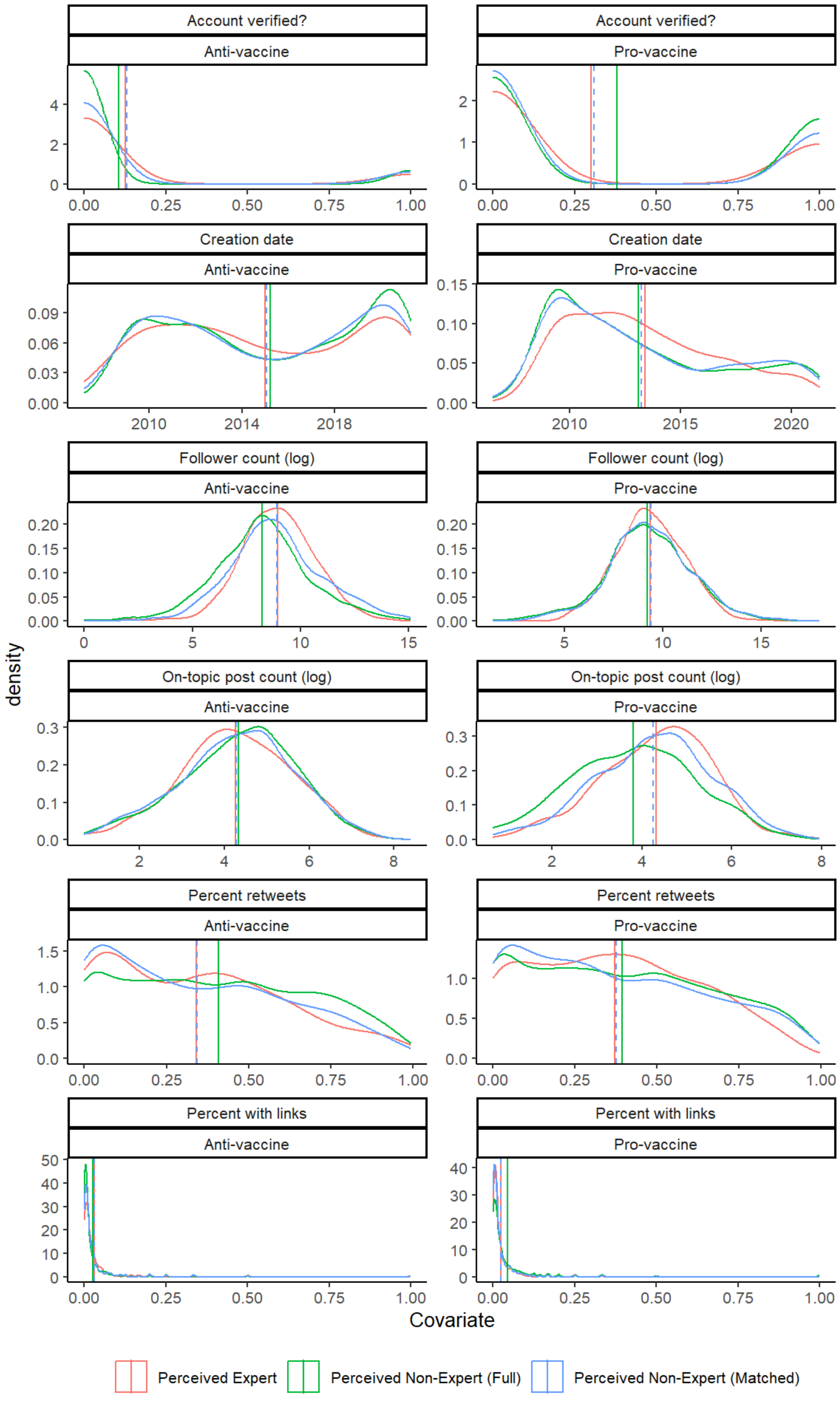

Figure 17: **Matching reduces differences in covariate distributions between perceived experts and perceived non-experts.** Caption continued on following page.

Figure 17: (continued) Each plot shows the frequency distribution of users for a given matching covariate (described in Supplemental Table 2). Lines are colored by the subset of users: perceived experts (red), perceived non-experts in the full community prior to matching (green), and perceived non-experts in the community subset for matching (blue); vertical lines indicate the mean value for the corresponding group of users. Each row corresponds to a different matching covariate: whether the account is verified, account creation date, logged follower count, logged count of on-topic posts, percent of posts that are retweets, and percent of posts with links. Each column corresponds to a different community: anti-vaccine (left) or pro-vaccine (right).

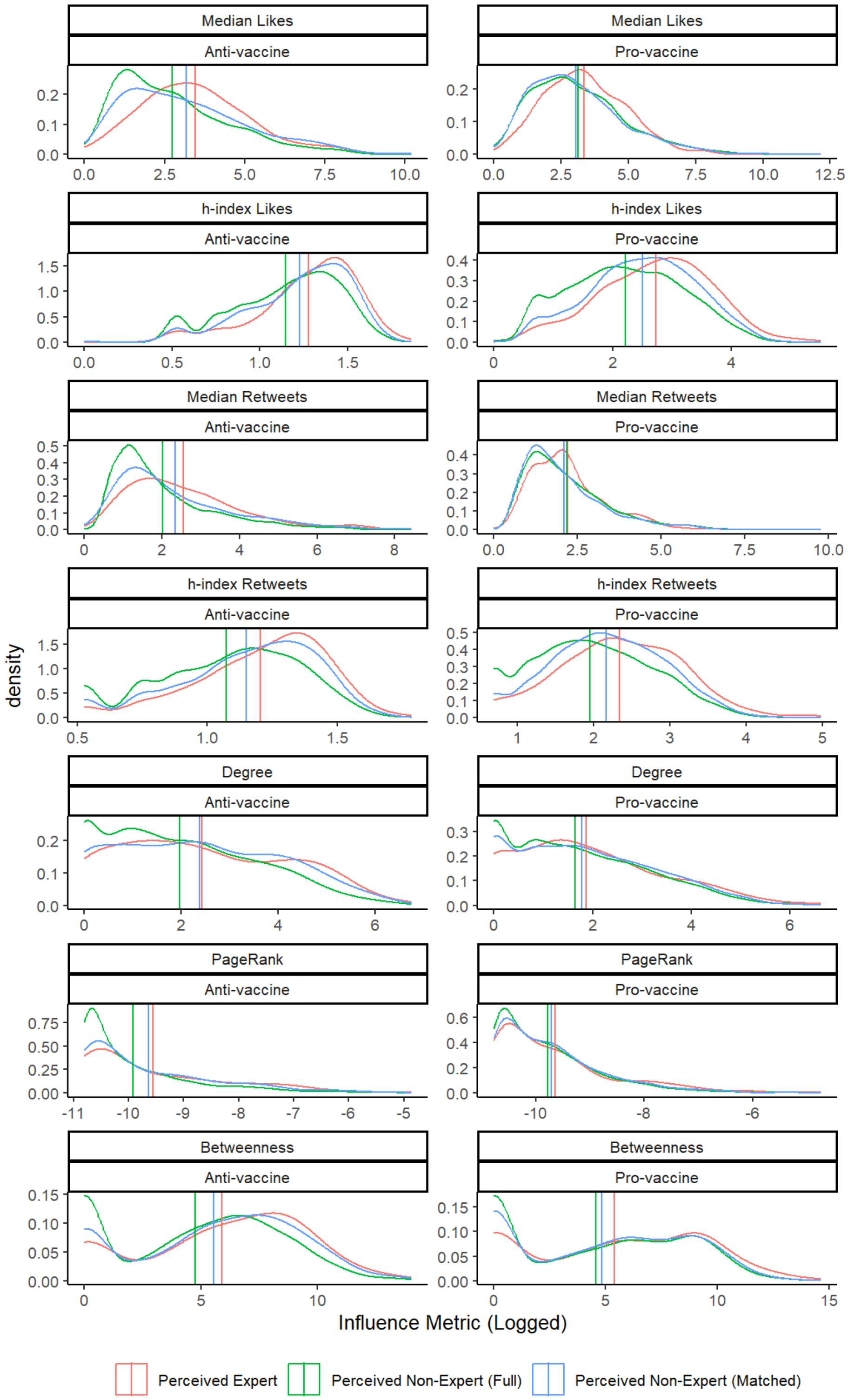

Figure 18: **Matching generally reduces the difference in engagement metrics between perceived experts and perceived non-experts.** Caption continued on following page.

Figure 18: (continued) Each plot shows the frequency distribution of users for a given natural logged influence metric (described in Supplemental Table 3). Lines are colored by the subset of users: perceived experts (red), perceived non-experts in the full community prior to matching (green), and perceived non-experts in the community subset for matching (blue); vertical lines indicate the mean value for the corresponding group of users. Each row corresponds to a different influence metric: median likes, h-index of likes, median retweets, h-index of retweets, degree, PageRank, and betweeenness. Each column corresponds to a different community: anti-vaccine (left) or pro-vaccine (right).

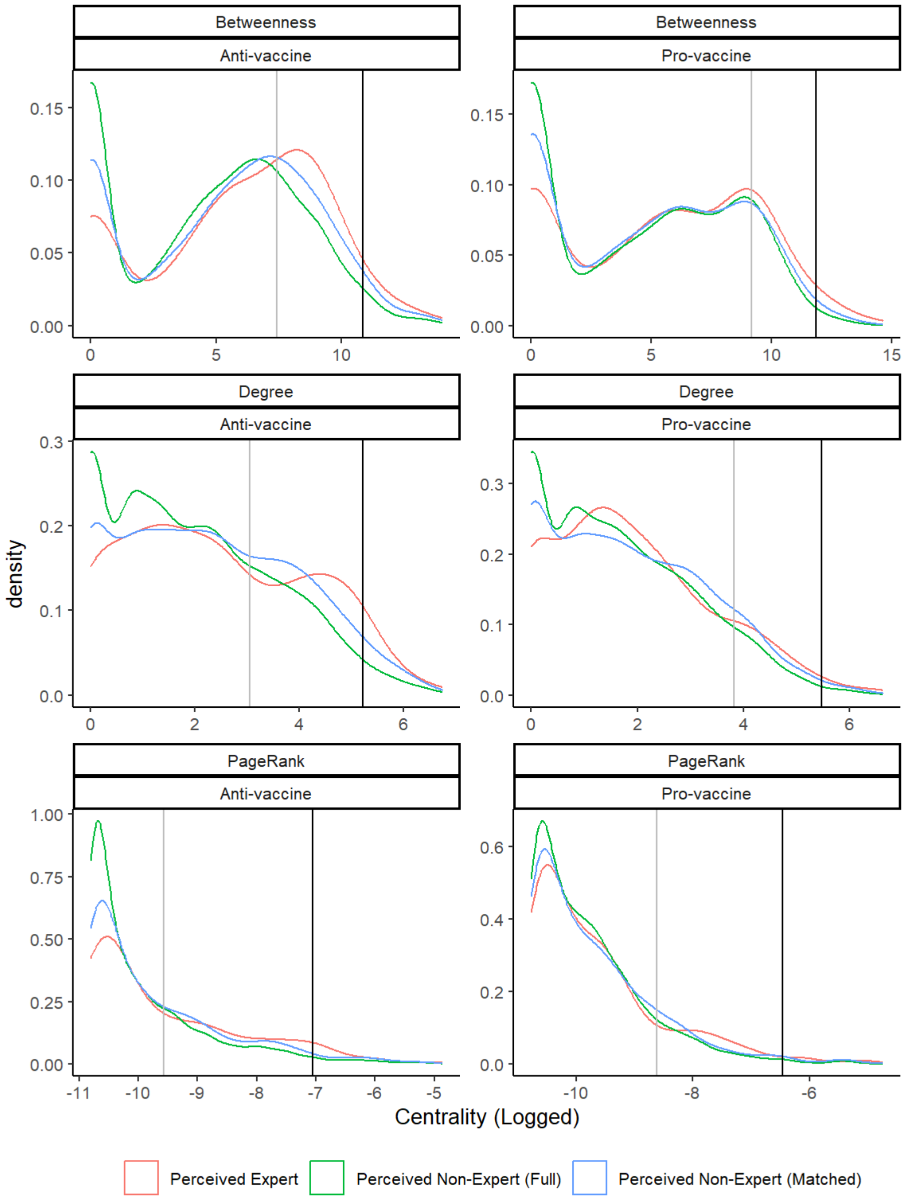

Figure 19: **Matching reduces the proportion of perceived non-experts with low centrality.** Each plot shows the frequency distribution of users for a given natural logged centrality metric. Lines are colored by the subset of users: perceived experts (red), perceived non-experts in the full community prior to matching (green), and perceived non-experts in the community subset for matching (blue). Values to the right of the vertical lines indicate users in the top 500 (gray) or 50 (black) users ranked by the given centrality metric. Each row corresponds to a different centrality metric: betweeenness (top), degree (middle), and PageRank (bottom). Each column corresponds to a different community: anti-vaccine (left) or pro-vaccine (right).

9 Sensitivity to matching specifications

This section shows that propensity score matching results are generally robust to k, the number of nearest neighbors to which each perceived expert is matched (Supplemental Figure 20), and the exclusion of any one matching covariates (Supplemental Figure 21 for balance, Supplemental Figure 22 for estimated ATT), with several exceptions. The finding that perceived expertise had a positive effect on betweenness and degree centrality in the pro-vaccine community did not hold when k was less than three. The significant effect of perceived expertise on median retweets in the anti-vaccine community did not hold when certain matching covariates were dropped (e.g., follower count, on-topic post count, percent retweets, percent with links) and when k was one. At the same time, in the pro-vaccine community, there was no positive effects of perceived expertise on degree centrality when certain covariates were dropped (e.g., account verification, on-topic post count, and posting time). There was a significantly greater effect of perceived expertise on median retweets in the anti-vaccine community compared to the pro-vaccine community when account verification status was dropped as a matching covariate.

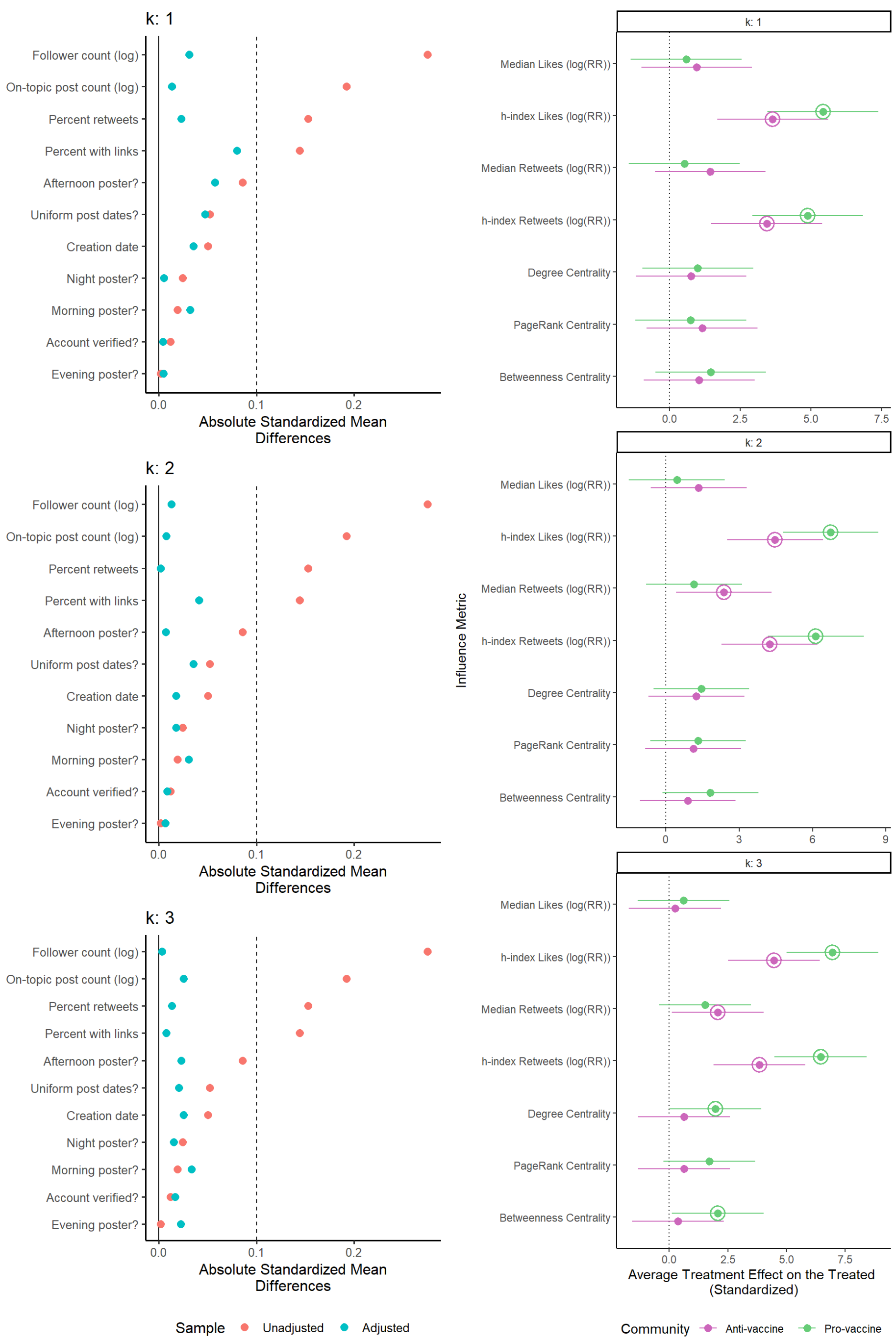

Figure 20: **Matching balance and results are generally robust to k, the number of nearest neighbors to which each perceived expert is matched.** Caption continued on following page.

Figure 20: (continued) Each row corresponds to a different value of k: 1 (top), 2 (middle), or 3 (bottom). The left column contains Love plots demonstrating the balance across matching covariates for individuals in the pro- and anti-vaccine communities before (orange) and after (blue) propensity score matching was performed. Within a plot, each row corresponds to a different matching covariate (described in Supplemental Table 2. The x-axis is absolute standardized mean difference, where values closer to zero correspond to better balance. The horizontal line indicates 0.1, the threshold below which balance is generally considered good. The right column shows the standardized average treatment effect on the treated (point) and corresponding 95 % confidence interval for the pro- (green) and anti-vaccine (pink) communities for each influence metric (y-axis, Supplemental Table 3). Positive values (to the right of the vertical line) indicate an influence boost for perceived experts. Instances where the average treatment effect on the treated was significantly greater than zero ($p < 0.05$) are indicated with an additional circle around the point estimate.

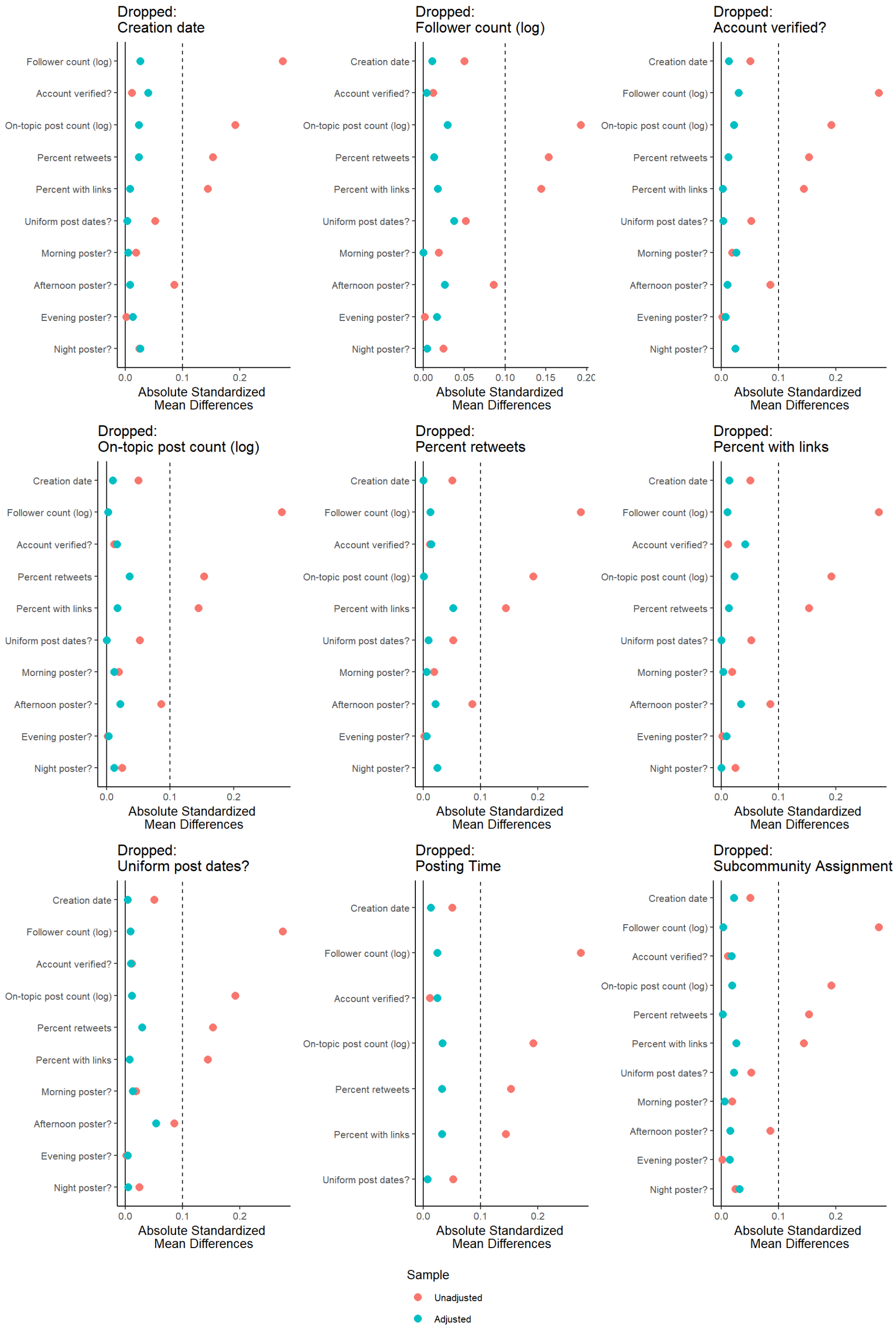

Figure 21: **Matching balance is robust to matching covariates.** Matching covariates were dropped one at a time to detect whether inclusion of a single covariate affected balance across other matching covariates. Caption continued on following page.

Figure 21: (continued) Each plot corresponds to a different dropped covariate indicated in the panel title (described in Supplemental Table 2). Note that the variable *posting time* encompasses three binary variables: *morning poster*, *evening poster*, and *night poster*. The variable *subcommunity assignment* encompasses four binary variables: *submodule 1-1*, *submodule 1-2*, *submodule 2-1*, and *submodule 2-1*. Each Love plots demonstrates the balance across matching covariates for individuals in the pro- and anti-vaccine communities before (orange) and after (blue) propensity score matching was performed. Each row within a figure corresponds to a different influence metric (Supplemental Table 3). The x-axis is absolute standardized mean difference, where values closer to zero correspond to better balance. The horizontal line indicates 0.1, the threshold below which balance is generally considered good.

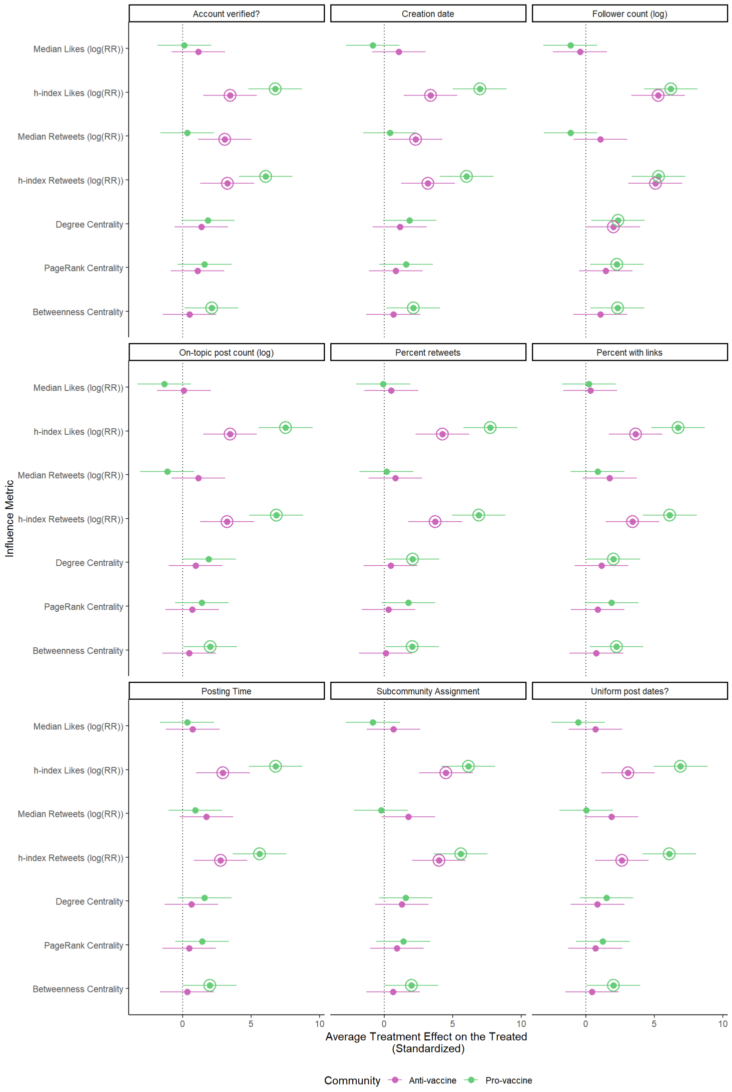

Figure 22: **Average treatment effect on the treated (ATT) is robust to matching covariates.** Matching covariates were dropped one at a time to detect whether inclusion of a single covariate affected ATT. Caption continued on following page.

Figure 22: (continued) Each plot corresponds to a different dropped matching covariate indicated in the panel title (described in Supplemental Table 2). Plots show the standardized average treatment effect on the treated (point) and corresponding 95 % confidence interval for the pro- (green) and anti-vaccine (pink) communities for each influence metric (y-axis, Supplemental Table 3). The variable *subcommunity assignment* encompasses four binary variables: *submodule 1-1*, *submodule 1-2*, *submodule 2-1*, and *submodule 2-1*. Positive values (to the right of the vertical line) indicate an influence boost for perceived experts compared to other individual users. Instances where the average treatment effect on the treated was significantly greater than zero ($p < 0.05$) are indicated with an additional circle around the point estimate.

10 Sensitivity to coengagement network parameters

Here, we examine the sensitivity of results to the parameters used to construct coengagement network. In the main text, edges connected users who were retweeted at least ten times by at least two other accounts, emphasizing the behavior of a small set of highly engaged accounts (*n2s10*). We present results for two alternative parameter sets: edges based on at least two retweets from at least ten other accounts (emphasizing activity on more viral content, *n10s2*) and edges based on at least five retweets from at least five other accounts (an intermediate between the two other parameter sets, *n5s5*).

In all cases, there are two main communities: anti-vaccine and pro-vaccine, and there is substantial overlap in the users included in the different coengagement networks (Supplemental Figure 23). However, only about 50% of the users in the anti- and pro-vaccine communities for the n10s2 network were included in the main text analysis (Supplemental Figure 23). In Supplemental Figure 24 and Supplemental Figure 25, we visualize the n5s5 and n10s2 coengagement networks based on Infomap community assignment, noting that these networks appear more dense than those in the main text. In Supplemental Figure 26 and Supplemental Figure 27, we again visualize both coengagement networks but color nodes based on community assignment in the main text to enable comparison to Main Text Figure 1. As in Supplemental Figure 23, we see that there is substantial overlap in community assignment for users across coengagement network parameters.

We repeated the analyses in the main text to compare link-sharing, centrality, and engagements depending on community and perceived expertise. For both coengagement networks, we again find that users in the anti-vaccine community shared significantly more low quality links, regardless of perceived expertise, and perceived experts shared significantly more academic links, regardless of community (Supplemental Figure 28 and Supplemental Figure 29 for the n5s5 and n10s2 networks respectively, as compared to Main Text Figure 2). Generally, perceived experts were overrepresented as highly central users regardless of centrality metric, community, and coengagement network parameters, although the significance of this finding varied somewhat (Supplemental Figure 30 and Supplemental Figure 31 for the n5s5 and n10s2 networks respectively, as compared to Main Text Figure 3). Notably, perceived experts in the pro-vaccine community were not overrepresented as highly central when ranked by PageRank for the n5s5 network. Across all coengagement network parameters, perceived experts were highly overrepresented as the top 500, 50, and 10 bridges between the anti- and pro-vaccine communities (Supplemental Figure 32 and Supplemental Figure 33 for the n5s5 and n10s2 networks respectively, as compared to Main Text Figure 4).

Next, we performed propensity score matching on both coengagmeent networks, first noting that balance was achieved across all covariates regardless of coengagement network parameters (Supplemental Figure 34 and Supplemen-

tal Figure 35 for the n5s5 and n10s2 networks respectively, as compared to Supplemental Figure 15). The primary findings from the main text based on propensity score matching are generally robust: perceived experts receive significantly more engagements in both the anti- and pro-vaccine communities based on h-index metrics (Supplemental Figure 36 and Supplemental Figure 37 for the n5s5 and n10s2 networks respectively, as compared to Main Text Figure 5), and there is usually not a significant difference in the size of the influence boost for perceived experts between the two communities (Supplemental Figure 38 and Supplemental Figure 39 for the n5s5 and n10s2 networks respectively, as compared to Supplemental Figure 16)). However, there were some differences in which effects were significant for n5s5 and n10s2 compared to the main text coengagement network. There was no positive effect of perceived expertise in the anti-vaccine community on h-index retweets in n10s2, but there was a significant positive effect of perceived expertise in the anti-vaccine community on median likes for n5s5. In the pro-vaccine community, there was no significant effect of perceived expertise on betweenness centrality for n5s5 and no significant effect of perceived expertise on degree centrality for n5s5 and n10s2. In the n5s5 and n10s2 coengagement networks, there was also a significantly greater positive effect of perceived expertise on median retweets in the anti-vaccine community compared to the pro-vaccine community, providing some support for H3 (Supplemental Figure 38). We conclude that the main text findings are robust to different coengagement network parameters.

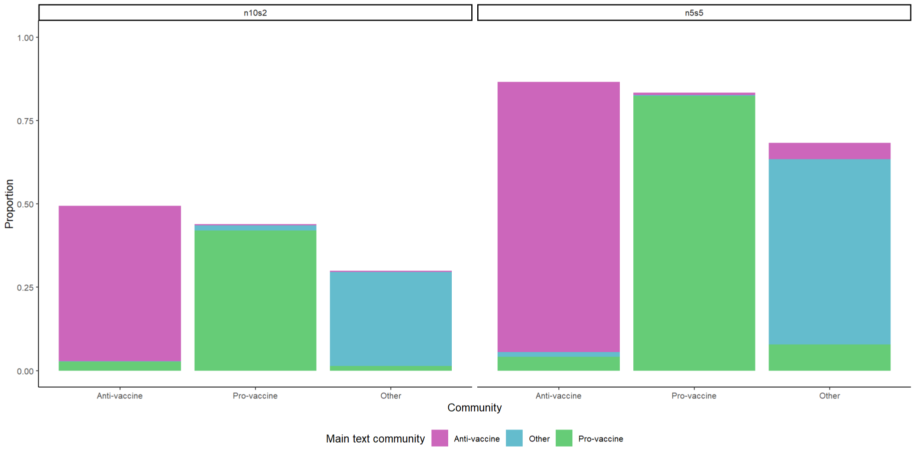

Figure 23: **Comparing overlap between main text coengagement network and comparison networks: n5s5 (left) and (n10s2).** Communities other than the largest two communities (anti-vaccine and pro-vaccine) are labelled "other". Each panel shows the proportion of users assigned to a given community in n5s5 or n10s2 (x-axis: anti-vaccine, pro-vaccine, or other) compared to the community they were assigned in the main text (colors: pink (anti-vaccine), green (pro-vaccine), or blue (other)). All other users were not in the main text coengagement network.

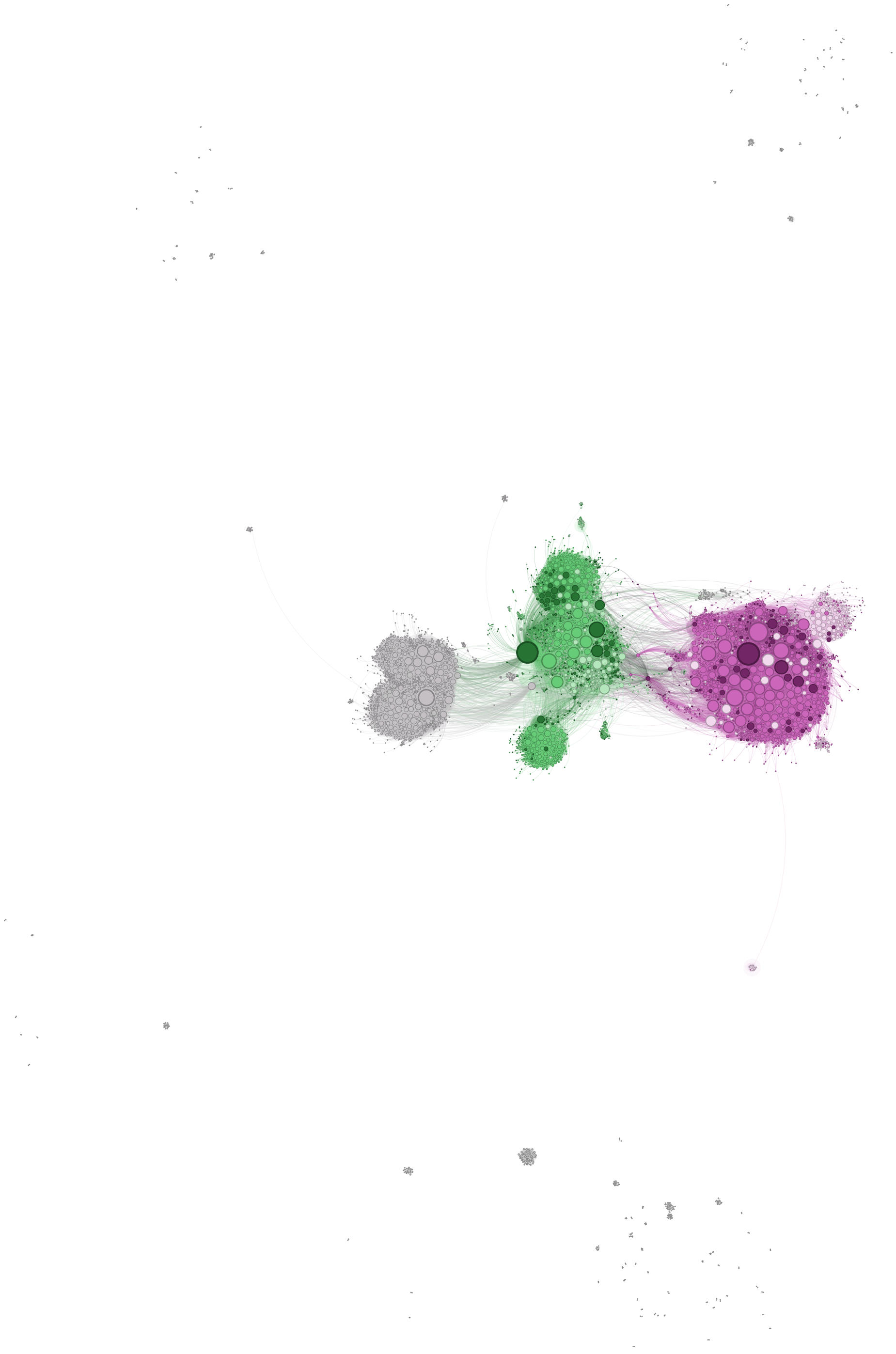

Figure 24: **The n5s5 coengagement network.** Nodes in the two largest communities detected using the Infomap algorithm are colored in pink (anti-vaccine) and green (pro-vaccine). Shades indicate account type: non-individual and non-English accounts excluded from analyses (light); perceived non-expert (medium); and perceived experts (dark). Nodes outside of the two largest communities are gray. Each edge is colored based on the color of one of the two nodes it connects, randomly selected. This figure may be compared to Main Text Figure 1.

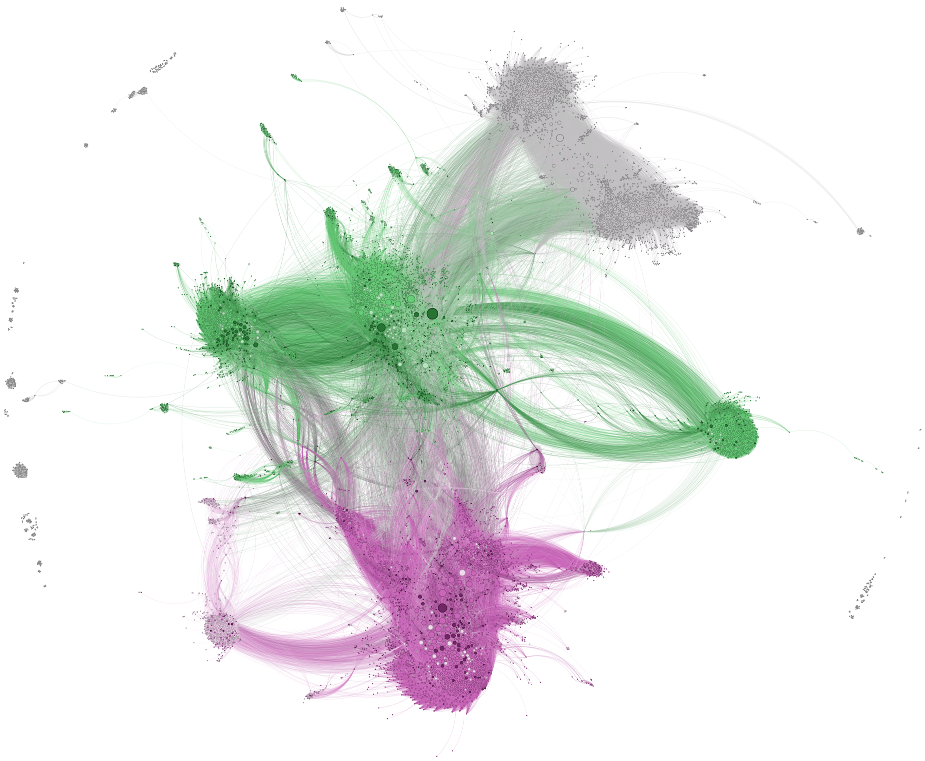

Figure 25: **The n10s2 coengagement network.** Nodes in the two largest communities detected using the Infomap algorithm are colored in pink (anti-vaccine) and green (pro-vaccine). Shades indicate account type: non-individual and non-English accounts excluded from analyses (light); perceived non-expert (medium); and perceived experts (dark). Nodes outside of the two largest communities are gray. Each edge is colored based on the color of one of the two nodes it connects, randomly selected. This figure may be compared to Main Text Figure 1.

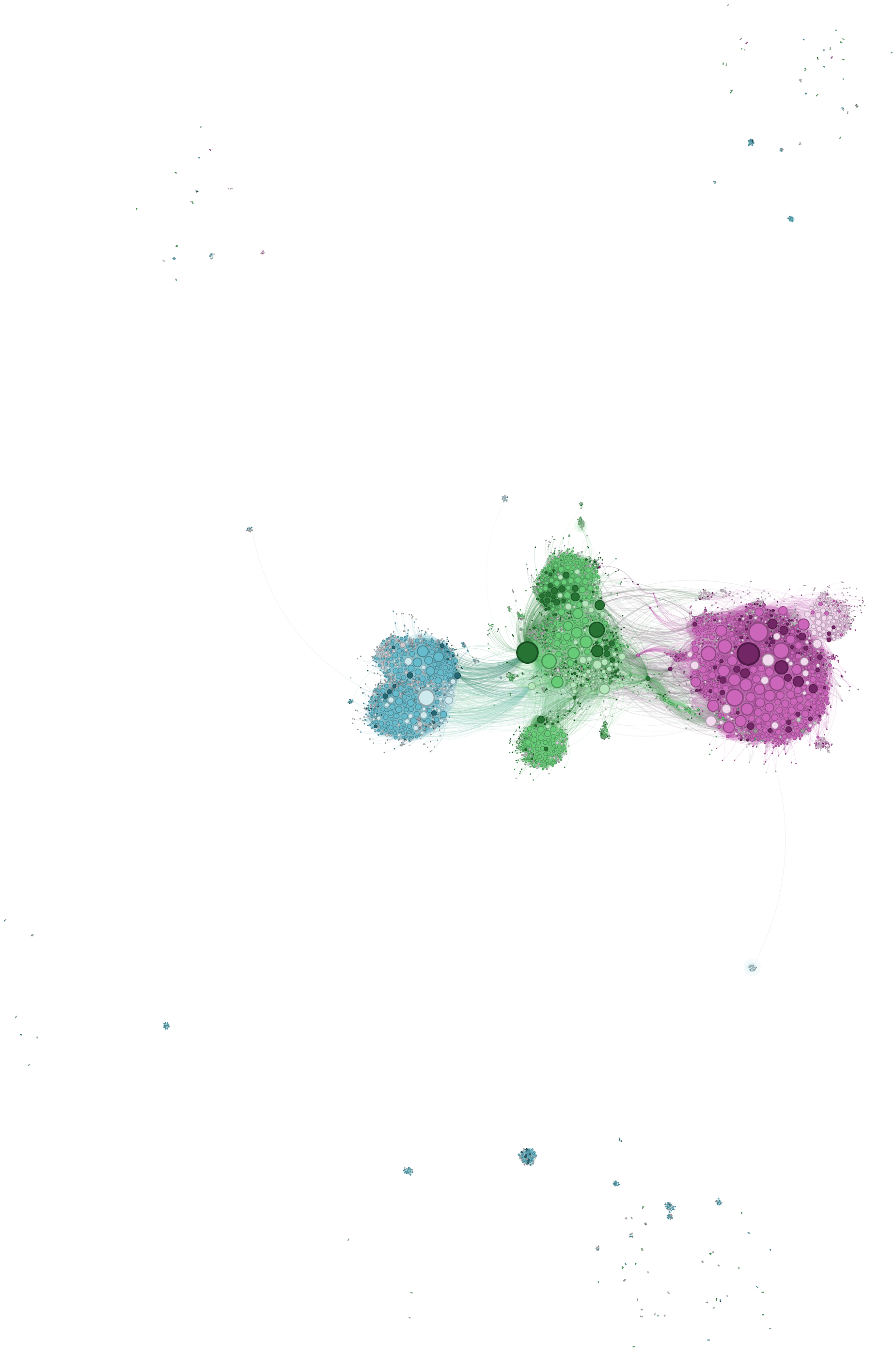

Figure 26: **Comparison of the n5s5 coengagement network to the coengagement network in the main text.** Users are represented as circles and scaled by degree centrality. Nodes that are also in the main text coengagement network are colored based on their community in that coengagement network: anti-vaccine (pink), pro-vaccine (green), or other (blue). Nodes that were not in the main text conegagement network are indicated in gray. Shades indicate account type: non-individual and non-English accounts excluded from analyses (light); perceived non-expert (medium); and perceived experts (dark). Each edge is colored based on the color of one of the two nodes it connects, randomly selected. This figure may be compared to Main Text Figure 1.

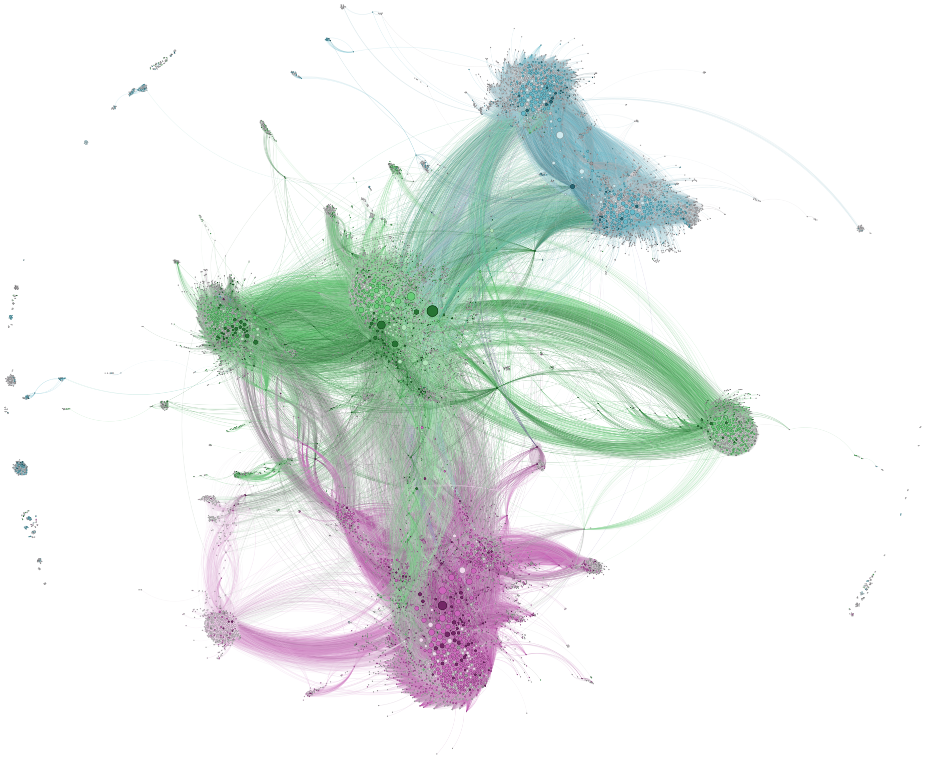

Figure 27: **Comparison of the n10s2 coengagement network to the coengagement network in the main text.** Users are represented as circles and scaled by degree centrality. Nodes that are also in the main text coengagement network are colored based on their community in that coengagement network: anti-vaccine (pink), pro-vaccine (green), or other (blue). Nodes that were not in the main text conegagement network are indicated in gray. Shades indicate account type: non-individual and non-English accounts excluded from analyses (light); perceived non-expert (medium); and perceived experts (dark). Each edge is colored based on the color of one of the two nodes it connects, randomly selected. This figure may be compared to Main Text Figure 1.

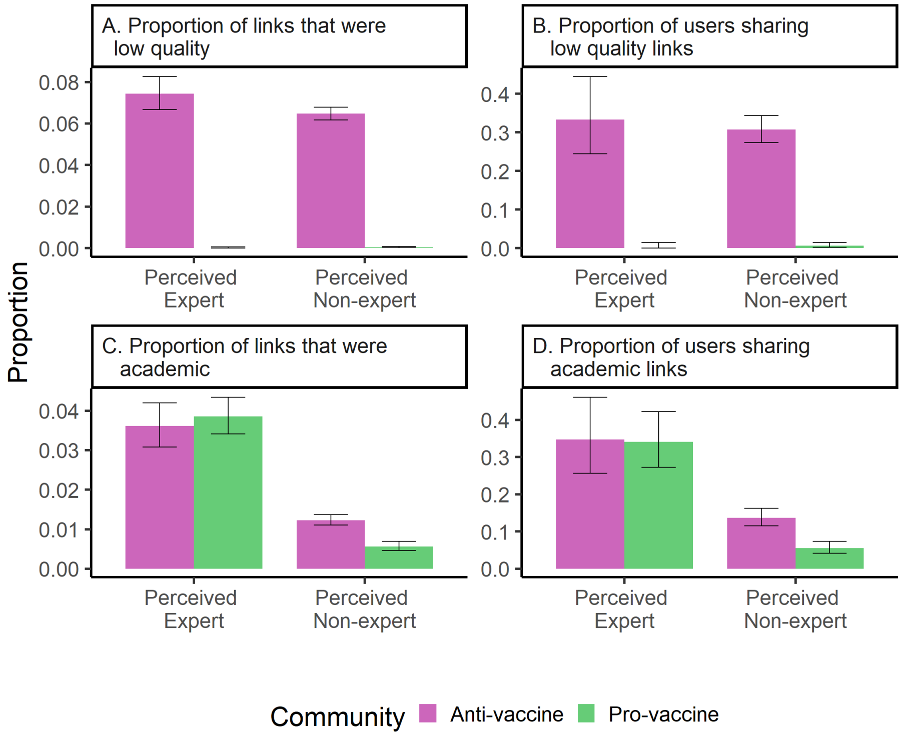

Figure 28: **Link analysis for the n5s5 coengagement network.** Each panel compares a different metric of link-sharing by perceived experts and perceived non-experts in the anti- (pink) and pro- (green) vaccine communities. The metrics are: (A) proportion of checked links that were from low quality sources, (B) proportion of users that shared at least one low quality source, (C) proportion of checked links that were from academic research sources, and (D) proportion of users that shared at least one academic research source. 95% binomial proportion confidence intervals are indicated by black error bars.

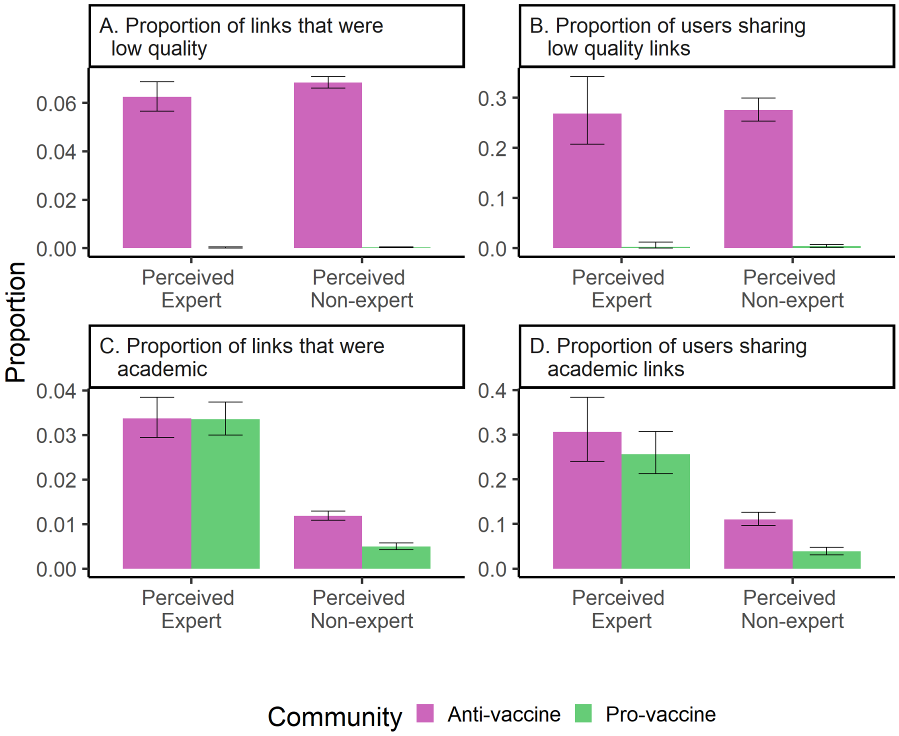

Figure 29: **Link analysis for the n10s2 coengagement network.** Each panel compares a different metric of link-sharing by perceived experts and perceived non-experts in the anti- (pink) and pro- (green) vaccine communities. The metrics are: (A) proportion of checked links that were from low quality sources, (B) proportion of users that shared at least one low quality source, (C) proportion of checked links that were from academic research sources, and (D) proportion of users that shared at least one academic research source. 95% binomial proportion confidence intervals are indicated by black error bars.

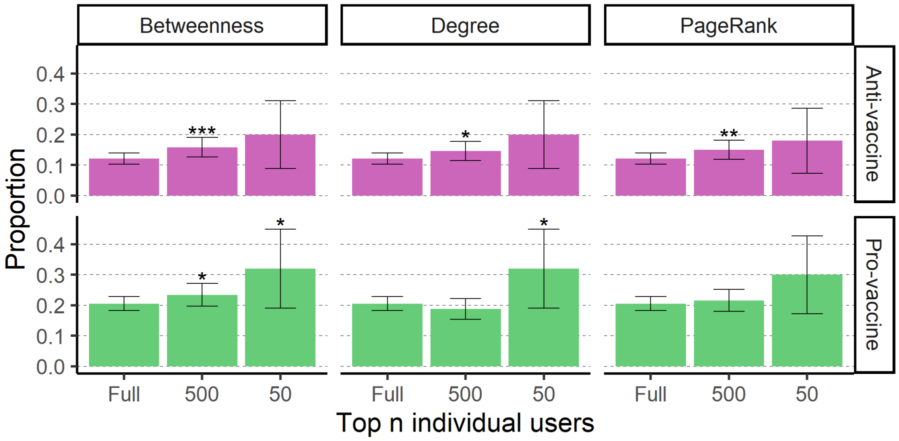

Figure 30: **Perceived experts are overrepresented as the most central users in the n5s5 coengagement network.** Plots are arranged in a grid where each row corresponds to users in one of the two largest communities: the anti- and pro-vaccine communities (top and bottom respectively). Each column corresponds to a different centrality metric: betweenness centrality (left), degree centrality (middle), and PageRank centrality (right). For each plot, we subset the network to the n users with the greatest values for a given centrality metric, where n is full population size (all individual users in the community), 500, or 50 (x-axis). Bar height indicates the proportion of users in each subset that are perceived experts and error bars give 95% binomial proportion confidence intervals. Stars above the error bars indicate whether perceived experts are significantly overrepresented within a given sample of central users (one star indicates $p < 0.05$, two stars indicate $p < 0.01$, three stars indicate $p < 0.001$).

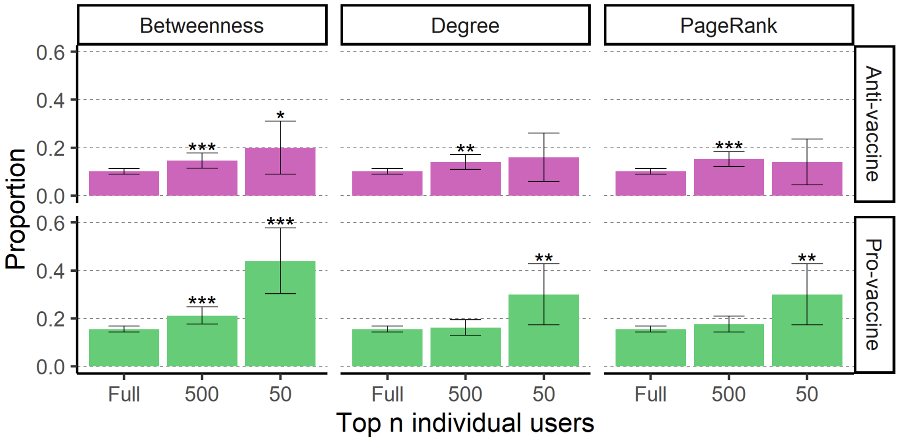

Figure 31: **Perceived experts are overrepresented as the most central users in the n102 coengagement network.** Plots are arranged in a grid where each row corresponds to users in one of the two largest communities: the anti- and pro-vaccine communities (top and bottom respectively). Each column corresponds to a different centrality metric: betweenness centrality (left), degree centrality (middle), and PageRank centrality (right). For each plot, we subset the network to the n users with the greatest values for a given centrality metric, where n is full population size (all individual users in the community), 500, or 50 (x-axis). Bar height indicates the proportion of users in each subset that are perceived experts and error bars give 95% binomial proportion confidence intervals. Stars above the error bars indicate whether perceived experts are significantly overrepresented within a given sample of central users (one star indicates $p < 0.05$, two stars indicate $p < 0.01$, three stars indicate $p < 0.001$).

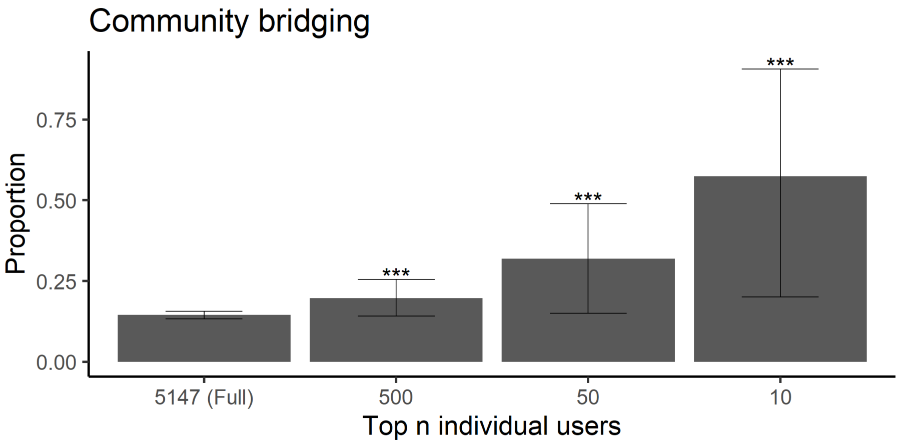

Figure 32: **Perceived experts are overrepresented as key bridges between the pro- and anti-vaccine communities in the n5s5 coengagement network.** Bar height indicates the proportion of users in each population sample that are perceived experts and error bars give 95% confidence intervals for the proportions. The x-axis indicates the size of each subset (n), corresponding to the full population (all individual users as a basis of comparison), and the 500, 50, or 10 users with the greatest community bridging score (x-axis). Stars above the error bars indicate whether perceived experts are significantly overrepresented within a given sample of bridging users (one star indicates $p < 0.05$, two stars indicate $p < 0.01$, three stars indicate $p < 0.001$).

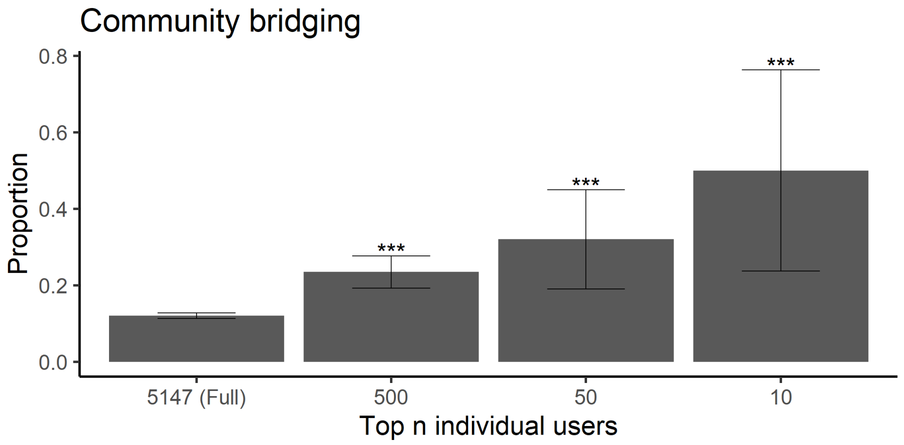

Figure 33: **Perceived experts are overrepresented as key bridges between the pro- and anti-vaccine communities in the the n10s2 co-engagement network.** Bar height indicates the proportion of users in each population sample that are perceived experts and error bars give 95% confidence intervals for the proportions. The x-axis indicates the size of each subset (n), corresponding to the full population (all individual users as a basis of comparison), and the 500, 50, or 10 users with the greatest community bridging score (x-axis). Stars above the error bars indicate whether perceived experts are significantly overrepresented within a given sample of bridging users (one star indicates $p < 0.05$, two stars indicate $p < 0.01$, three stars indicate $p < 0.001$).

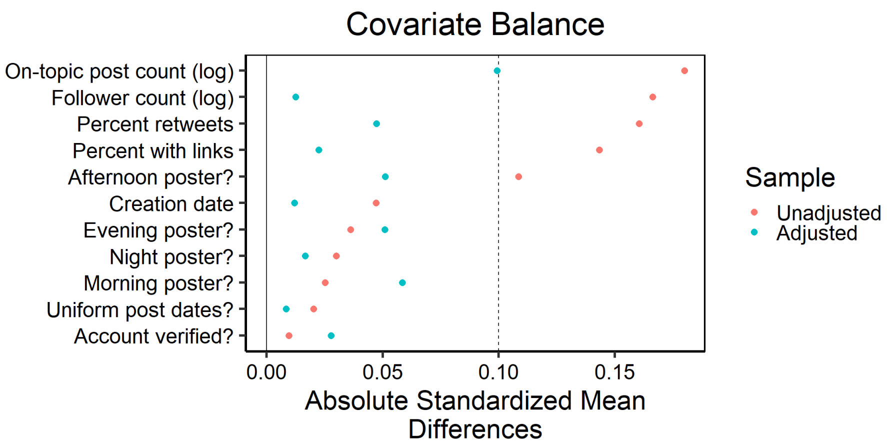

Figure 34: **Love plot demonstrating the balance across matching covariates in the n5s5 coengagement network.** We compare balance in the pro- and anti-vaccine communities before (orange) and after (blue) propensity score matching was performed to test H3. Each row corresponds to a different matching covariate (described in Supplemental Table 2). The x-axis is absolute standardized mean difference, where values closer to zero correspond to better balance. The horizontal line indicates 0.1, the threshold below which balance is generally considered good.

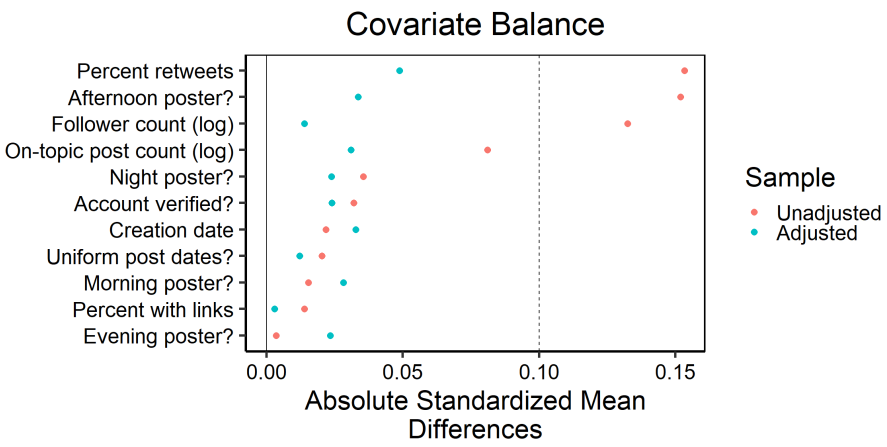

Figure 35: **Love plot demonstrating the balance across matching covariates in the n10s2 coengagement network.** We compare balance in the pro- and anti-vaccine communities before (orange) and after (blue) propensity score matching was performed to test H3. Each row corresponds to a different matching covariate (described in Supplemental Table 2). The x-axis is absolute standardized mean difference, where values closer to zero correspond to better balance. The horizontal line indicates 0.1, the threshold below which balance is generally considered good.

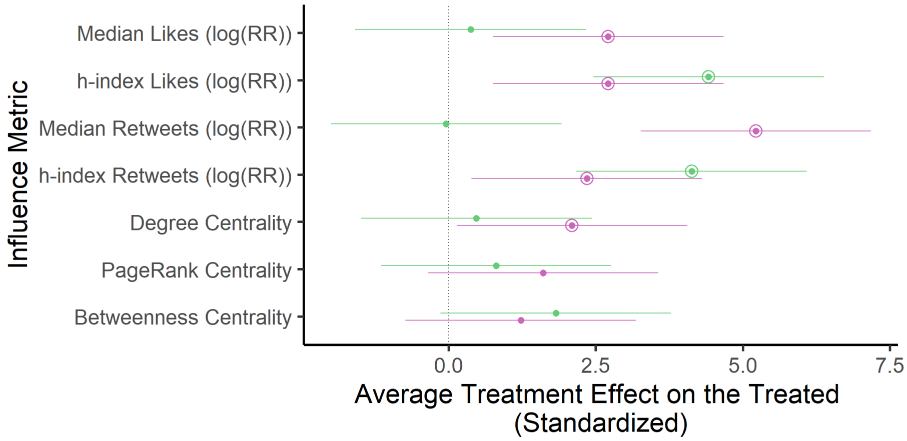

Figure 36: **In the anti-vaccine and pro-vaccine communities, perceived experts receive greater engagements compared to other users in the n5s5 coengagement network.** Plots show the standardized average treatment effect on the treated (point) and corresponding 95 % confidence interval for the pro- (green) and anti-vaccine (pink) communities for each influence metric (y-axis, Supplemental Table 3). Positive values (to the right of the vertical line) indicate an influence boost for perceived experts. Instances where the effects were significantly greater than zero ($p < 0.05$) are indicated with an additional circle around the point estimate.

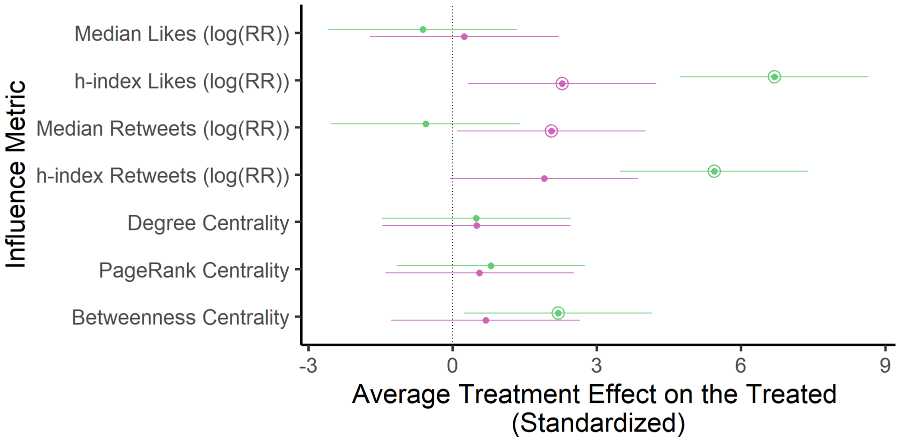

Figure 37: **In the anti-vaccine and pro-vaccine communities, perceived experts receive greater engagements compared to other users in the n10s2 coengagement network.** Plots show the standardized average treatment effect on the treated (point) and corresponding 95 % confidence interval for the pro- (green) and anti-vaccine (pink) communities for each influence metric (y-axis, Supplemental Table 3). Positive values (to the right of the vertical line) indicate an influence boost for perceived experts. Instances where the effects were significantly greater than zero ($p < 0.05$) are indicated with an additional circle around the point estimate.

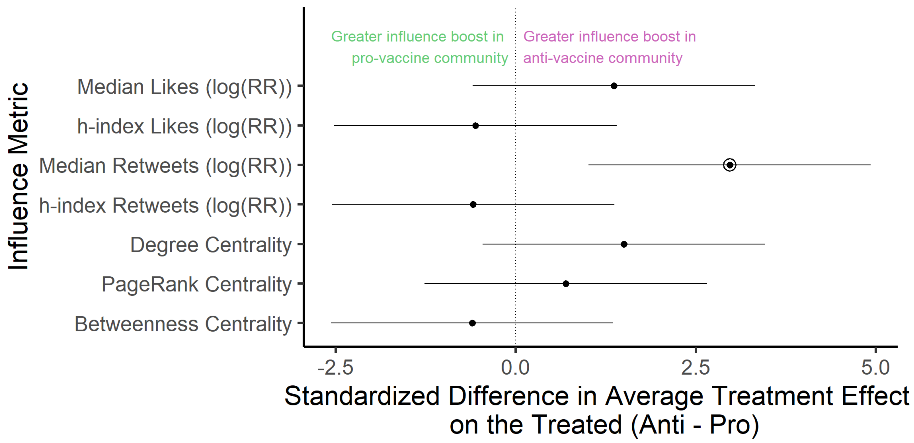

Figure 38: **There is a significant difference in the influence boost for perceived experts on median retweets between the anti- and pro-vaccine communities for users in the n5s5 coengagement network.** For each influence metric (y-axis, Supplemental Table 3, we plot the difference in the standardized average treatment effect on the treated (ATT) between the pro- and anti-vaccine communities as a point and corresponding 95% confidence interval. Positive values (to the right of the vertical line) indicate a greater influence boost for perceived experts in the anti-vaccine commmunity compared to the pro-vaccine community. Instances where the effects were significantly greater than zero ($p < 0.05$) are indicated with an additional circle around the point estimate.

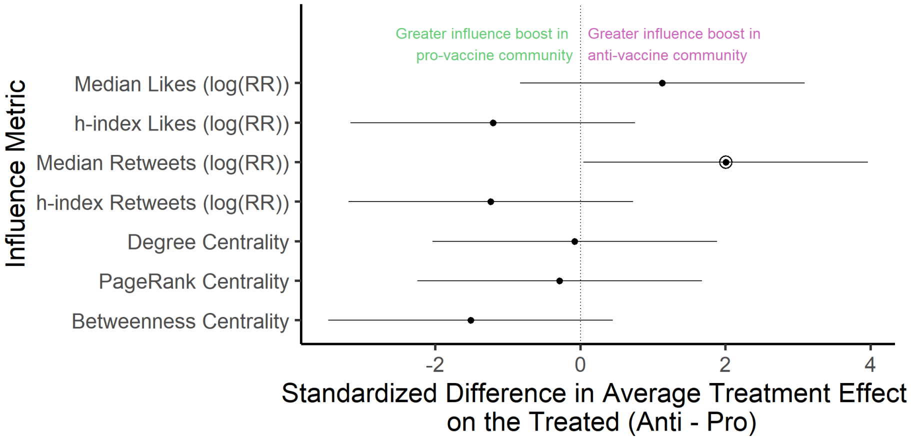

Figure 39: **There is a significant difference in the influence boost for perceived experts on median retweets between the anti- and pro-vaccine communities for users in the n2s10 coengagement network.** For each influence metric (y-axis, Supplemental Table 3, we plot the difference in the standardized average treatment effect on the treated (ATT) between the pro- and anti-vaccine communities as a point and corresponding 95% confidence interval. Positive values (to the right of the vertical line) indicate a greater influence boost for perceived experts in the anti-vaccine commmunity compared to the pro-vaccine community. Instances where the effects were significantly greater than zero ($p < 0.05$) are indicated with an additional circle around the point estimate.

References

A. Beers, J. S. Schafer, I. Kennedy, M. Wack, E. S. Spiro, and K. Starbird. Followback Clusters, Satellite Audiences, and Bridge Nodes: Coengagement Networks for the 2020 US Election. *Proceedings of the International AAAI Conference on Web and Social Media*, June 2023.

J.-C. Boucher, K. Cornelson, J. L. Benham, M. M. Fullerton, T. Tang, C. Constantinescu, M. Mourali, R. J. Oxoby, D. A. Marshall, H. Hemmati, A. Badami, J. Hu, and R. Lang. Analyzing Social Media to Explore the Attitudes and Behaviors Following the Announcement of Successful COVID-19 Vaccine Trials: Infodemiology Study. *JMIR Infodemiology*, 1(1):e28800, Aug. 2021. ISSN 2564-1891. doi: 10.2196/28800. URL https://infodemiology.jmir.org/2021/1/e28800.

L. Hagen, A. Fox, H. O'Leary, D. Dyson, K. Walker, C. A. Lengacher, and R. Hernandez. The Role of Influential Actors in Fostering the Polarized COVID-19 Vaccine Discourse on Twitter: Mixed Methods of Machine Learning and Inductive Coding. *JMIR Infodemiology*, 2(1):e34231, June 2022. ISSN 2564-1891. doi: 10.2196/34231. URL https://infodemiology.jmir.org/2022/1/e34231.

A. Holmgren, D. Edler, and M. Rosvall. Infomap Online, 2022. URL https://mapequation.org/infomap.

M. Roy. U.S. CDC expands COVID-19 vaccine eligibility to 16 and older. *Reuters*, Apr. 2021. URL https://www.reuters.com/world/us/us-cdc-expands-covid-19-vaccine-eligibility-16-older-2021-04-20/.

The Virality Project. Virality Project Weekly Briefing. Technical Report 17, Apr. 2021. URL https://www.viralityproject.org/s/Virality-Project-April-20-Weekly-Briefing-2.pdf.

U.S. Food and Drug Administration. FDA and CDC Lift Recommended Pause on Johnson & Johnson (Janssen) COVID-19 Vaccine Use Following Thorough Safety Review, Apr. 2021. URL https://www.fda.gov/news-events/press-announcements/fda-and-cdc-lift-recommended-pause-johnson-johnson-janssen-covid-19-vaccine-use-following[illegible]
